# Effects of *Inonotus obliquus* on ameliorating podocyte injury in ORG mice through TNF pathway and prediction of active compounds

**DOI:** 10.3389/fphar.2024.1426917

**Published:** 2024-08-21

**Authors:** Zhaodi Han, Le Gong, Yani Xue, Rui Wang, Jing Liu, Xinyu Wang, Wenyan Zhao, Hui Liao, Rongshan Li

**Affiliations:** ^1^ Drug Clinical Trial Institution, Shanxi Provincial People’s Hospital Affiliated to Shanxi Medical University, Taiyuan, China; ^2^ School of Pharmacy, Shanxi Medical University, Taiyuan, China; ^3^ Department of Nephrology, Shanxi Provincial People’s Hospital Affiliated to Shanxi Medical University, Taiyuan, China

**Keywords:** *Inonotus obliquus*, obesity-related glomerulopathy, podocyte injury, TNF signal, triterpenoids

## Abstract

**Background:**

Podocyte injury is a common pathologic mechanism in diabetic kidney disease (DKD) and obesity-related glomerulopathy (ORG). Our previous study confirmed that *Inonotus obliquus* (IO) improved podocyte injury on DKD rats. The current study explored the pharmacological effects, related mechanisms and possible active components of IO on ORG mice.

**Methods:**

Firstly, by combining ultra-high performance liquid chromatography tandem mass spectrometry analysis (UPLC-Q-TOF-MS) with network pharmacology to construct the human protein-protein interaction mechanism and enrich the pathway, which led to discover the crucial mechanism of IO against ORG. Then, ORG mice were established by high-fat diet and biochemical assays, histopathology, and Western blot were used to explore the effects of IO on obesity and podocyte injury. Finally, network pharmacology-based findings were confirmed by immunohistochemistry. The compositions of IO absorbed in mice plasma were analyzed by UPLC-Q-TOF-MS and molecular docking was used to predict the possible active compounds.

**Results:**

The network pharmacology result suggested that IO alleviated the inflammatory response of ORG by modulating TNF signal. The 20-week *in vivo* experiment confirmed that IO improved glomerular hypertrophy, podocyte injury under electron microscopy, renal nephrin, synaptopodin, TNF-α and IL-6 expressions with Western blotting and immunohistochemical staining. Other indicators of ORG such as body weight, kidney weight, serum total cholesterol, liver triglyceride also improved by IO intervention. The components analysis showed that triterpenoids, including inoterpene F and trametenolic acid, might be the pharmacodynamic basis.

**Conclusion:**

The research based on UPLC-Q-TOF-MS analysis, network pharmacology and *in vivo* experiment suggested that the amelioration of IO on podocyte injury in ORG mice via its modulation on TNF signal. Triterpenoids were predicated as acting components.

## 1 Introduction

*Inonotus obliquus* (IO) is a white rot fungus that has been used in traditional medicine for centuries, especially in Russia and northeastern China (Hao et al., 2023). Studies have shown that the main components of IO include triterpenoids, flavonoids, organic acids, polyphenols and alkaloids and other compounds, which have antioxidant, anti-inflammatory, hypoglycemic and other pharmacological effects (Ren et al., 2018; Anwaier et al., 2020). And, the diverse bioactive compounds confer IO great potential to inhibit podocyte injury and improve renal function (Duan et al., 2022). Our previous literature study showed that IO has been studied predominantly on cancer area, followed by diabetes mellitus and its complications (Shen et al., 2022). We further confirmed that IO delays the development of proteinuria in type 2 diabetic kidney disease (DKD) rat model through hypoglycemic, hypolipidemic, improving renal hemodynamics and other related effects (Zhang et al., 2022).

Podocyte injury in the glomerulus is central to the development of proteinuria in various chronic kidney diseases, including DKD (Liu S. et al., 2022), obesity-related glomerulopathy (ORG) (Wei et al., 2021), etc. Unlike DKD, which is clinically characterized by persistent hyperglycemia, obesity is recognized as an independent risk factor for the development of ORG (Sandino et al., 2022). Weight loss and proteinuria reduction are obviously the key to treat ORG (Mangat et al., 2023). Our previous work confirmed that amelioration of IO on proteinuria in DKD rats is associated with its reduction in podocyte injury, and we also observed that IO has a role in reducing body weight (BW) in DKD rats (Zhang et al., 2022). Based on our previous studies, we hypothesized that IO might have a protective effect for ORG.

Before performing an *in vivo* study on the above hypotheses, we first analyzed the possible mechanisms by which the components contained in IO intervene in ORG. As a useful tool, network pharmacology is believed to achieve a comprehensive insight into the therapeutical mechanism of multi-compound traditional medicine (Hao et al., 2023). Network pharmacology integrates cheminformatics, network topology and omics data, to holistically elucidate drug mechanisms (Muthuramalingam et al., 2023). Although studies have been conducted using a network pharmacology approach to investigate the mechanism of action of IO in the treatment of other diseases, there are still some limitations (Liu et al., 2021). One such limitation is that the information of IO components is derived from databases and published literature, which may not fully represent the complex of the real ingredient system.

Ultra-performance liquid chromatography coupled with electrospray tandem mass spectrometry (UPLC-Q-TOF-MS) is used in the qualitative and quantitative analysis of complex ingredient systems due to its powerful functions and wide applicability (Jiang et al., 2023). UPLC utilizes micron-sized stationary phases and higher column pressures to dramatically increase separation efficiency (Yang et al., 2024). ESI-Triple TOF MS/MS could perform multi-stage mass spectrometry detection of the target compounds to provide rich structural and quantitative information (Shu et al., 2023; Xue et al., 2024). Online mass spectrometry databases, such as Massbank, mzClound, could identify and confirm the components separated and detected by UPLC-Q-TOF-MS (Song et al., 2022). The integrated application of these techniques in this manuscript provided the basis for conducting component analysis, absorption, metabolism, and mechanism of IO.

A human protein-protein interactome (PPI) network for IO against ORG was constructed by integrating UPLC-Q-TOF-MS profiling and databases searching. Moreover, through network analysis for PPI and pathway enrichment, the key pathway and targets were focused. Subsequently, it was validated by establishing an ORG mice model and measuring BW, triglyceride (TG), total cholesterol (TC), renal function and podocyte protein expression after intervention with IO. Afterwards, the absorbed into blood components of IO were analyzed by UPLC-Q-TOF-MS, and combined with the results of network pharmacology, the pharmacodynamic components were proposed and verified by molecular docking. This provided a novel strategy for discovering the mechanism and active compounds of IO alleviating ORG.

## 2 Material and methods

### 2.1 Chemicals and reagents

IO was collected from Huzhong National Nature Reserve of the Greater Khingan Mountains in Heilongjiang Province. The traits of IO used in this study were spherical, oblate, or irregular mass, varying in size, 25–40 cm in diameter, sessile, and with a verrucose protrusion on the surface. The outer surface is gray-black with irregular deep furrowed wrinkled texture. The texture is hard, the outer layer of the section is thin, about 5 mm thick, dark brown, and the flesh is yellow to yellow-brown. The odor is slight, and the taste is slightly sweet. IO used in this study was identified as “*Inonotus obliquus* (Ach. ex Pers.) Pilát” (Dai and Li, 2011) by Professor Shang Guo (Shanxi Institute for Functional Food, Shanxi Agricultural University, Taiyuan, China).

Ordinary diet and high-fat-diet (HFD) were obtained from Boaigang Biotechnology Company (Beijing, China). The ordinary diet consists of 67.4% of calories from carbohydrates, 20.6% from protein and 12% from fat, and the HFD is 60% of calories from fat, 20% from protein and 20% from carbohydrates. The analytical grade organic solvents, such as methanol and acetonitrile, were bought from Thermo Fisher Scientific (USA).

### 2.2 Preparation of IO extracts and qualitative analysis by UPLC-Q-TOF-MS

The preparation of IO extracts was detailed in our previous study (Zhang et al., 2022). The sample of IO extract was mixed with 50% methanol-acetonitrile (1:10, w/v) and then ultrasonicated for 30 min. After sonication, the mixed sample was centrifuged (17,000 g, 15 min, 20°C) and the supernatant was taken in an EP tube which was then dried by vacuum at 35°C. The 100 μL of 50% methanol-water was added to the dried sample. After vortexing and centrifuging, the supernatant was analyzed by an UPLC system (Shimadzu, JPN).

The chromatographic separation was carried out on Waters XSelect HSS T3 XP column (2.1 × 100 mm, 1.8 μm, United States) with the mobile phase composed of 0.1% formic acid in water and 0.1% formic acid in acetonitrile under gradient elution. The AB 5600 Triple TOF mass spectrometer (AB SCIEX, United States) was capable of primary and secondary mass spectrometry data acquisition based on IDA functionality under Analyst TF 1.7, AB Sciex software control. In each acquisition cycle, the molecular ions with the maximum intensity and over 100 were selected for the acquisition of the corresponding secondary mass spectrometry data. MS detection range was m/z 50–1,200; electrospray bombardment energy was 30 eV; secondary mass spectra were acquired every 50 m. The ESI settings were as follows: gas pressure: 60 Psi; auxiliary air pressure: 60 Psi; capillary temperature, 350°C; in positive ion mode: spray voltage, 5 kV; in negative ion mode: spray voltage, 4 kV (Han et al., 2022).

The composition of the precursor and fragment ions were processed and sequenced by Analyst TF 1.7 software. After the data were converted to mzML format by Proteowizard software, MS-DIAL software was utilized for peak identification, peak filtration, and peak alignment, to obtain the data matrix, including m/z, intensity and retention time. Components identification was first confirmed on the basis of accurate molecular weight (Δ ≤ 30 ppm) and then further validation was based on the secondary mass spectrometry database (Massbank, GNPS, mzClound, etc.) as well as a self-constructed library of IO components constructed from previously published papers.

### 2.3 Collection of IO potential targets

The SMILE formula of IO components was imported into the SwissTargetPrediction platform (http://www.swisstargetprediction.ch) (Daina and Zoete, 2024), the species was set as “*Homo sapiens*” and the probability was set as “> 0.1.” Then the corresponding targets of IO components were obtained. The protein knowledgebase Uniprot (http://www.uniprot.org/) was employed to standardize the targets with restrict species setting as “*H. sapiens*.” The duplicate targets among the components were removed and potential targets of IO were obtained at last.

### 2.4 Collection of ORG therapeutic targets

The ORG therapeutic targets were assembled from a human gene database GeneCards (https://www.genecards.org/) (Stelzer et al., 2016), a database of online mendelian inheritance in man OMIM (https://www.omim.org) (McKusick, 2007) and a database of gene-disease associations DisGeNET (https://www.disgenet.org) (Piñero et al., 2020). In the above three databases, targets were obtained by searching the keywords “obesity-related glomerulopathy.” All the targets were pooled together and also standardized according to the information in Uniprot.

### 2.5 Collection of IO against ORG targets

The targets obtained in 2.3 and 2.4 were intercrossed by Bioinformatics & Evolutionary Genomics (https://bioinformatics.psb.ugent.be/webtools/Venn/) platform, and the therapeutic targets of IO components for ORG diseases were obtained.

### 2.6 Construction of PPI network

The targets obtained in 2.5 were uploaded into String database (https://cn.string-db.org/) (Szklarczyk et al., 2023). The species were also set as “*H. sapiens*” and the protein interaction score was set as “0.4.” Finally, the target interaction relationships under the human PPI background network were obtained. The results were imported into Cytoscape 3.8.2 software to construct the corresponding PPIs and the degree value of the target was calculated.

### 2.7 Pathway enrichment analysis

The targets obtained in 2.5 were subjected to Gene Ontology (GO) biological process (BP) enrichment analysis and signaling pathway Kyoto Encyclopedia of Genes and Genomes (KEGG) enrichment analysis using the Enrichr (https://maayanlab.cloud/Enrichr/) (Chen et al., 2013; Kuleshov et al., 2016; Xie et al., 2021), followed by visualization and analysis through the bioinformatics platform (https://www.bioinformatics.com.cn/) (Tang et al., 2023).

### 2.8 Animal experiments of IO treatment for ORG mice

Male C57BL/6J mice (4 weeks old) were purchased from Shanxi Provincial Key Laboratory of Nephrology and the Laboratory Animal Center (Taiyuan, China) and kept under specific pathogen free conditions (12-h light/dark cycle, 25°C ± 2°C) with free access to food and water. Animal experiments was approved by the Ethics Review Committee for Animal Experimentation of Shanxi Provincial People’s Hospital (Approval No. 2021-306). Animals were randomized divided into two groups after 1 week of acclimatization feeding: normal control group (CON with ordinary food, n = 6) and obesity model group (with HFD, n = 18).

The mice were weighed once a week. The model mouse was recognized to be obesity when its body weight ratio (BWR) exceeded 20% of CON in the same week (Wang Y. et al., 2023).

$$\text{The BWR }\left(\%\right)=\frac{\text{The}\;\text{BW}\;\text{of}\;\text{mouse}-\text{The}\;\text{average}\;\text{BW}\;\text{of}\;\text{CON}\;\text{group}}{\text{The}\;\text{average}\;\text{BW}\;\text{of}\;\text{CON}\;\text{group}}\times 100$$

Next, the obesity model group was randomly divided into three groups: the ORG model group (ORG, n = 6), the IO low-dose group (IO_L, n = 6), and the IO high-dose group (IO_H, n = 6). The mice in ORG group, IO_L group and IO_H group were continued to be fed with HFD until the end of the experiment. In addition, the mice in IO_L group and IO_H group were given IO extracts by gavage in a dose of 75 mg/kg/day and 150 mg/kg/day separately, for 12 consecutive weeks.

After the above four groups were identified, 24 h urine was collected and recorded from each group of mice by metabolic cages at weeks 7, 10, 12, 16, and 20, and the concentration of urinary proteins was determined by the Coomassie Brilliant Blue method. At week 18, intraperitoneal glucose tolerance test (IPGTT) and insulin tolerance test (IPITT) assays were performed (More details are provided in Supplementary Methods.

At the end of the experiment, blood, bilateral kidney and liver tissues were taken. Blood was centrifuged (3,000 rpm, 15 min, 4°C) to obtain serum, which were used to measure the levels of fasting blood glucose (FBG), serum creatinine (SCr), blood urea nitrogen (BUN) (Jiangsu Meimian Industrial Co. Ltd., China) and TC (Nanjing Jiancheng Bioengineering Institute, China). Liver tissues were used for histopathological examination and homogenized for determination of TG content with GPO-PAP kit assay (Nanjing Jiancheng Bioengineering Institute, China). Kidney tissues were weighed and used for histopathological examination and Western blot experiments.

### 2.9 Pathological examination

#### 2.9.1 Light microscopy

Freshly dissected renal and liver tissues were fixed in 10% neutral formalin buffer and embedded in paraffin. The embedded tissues were cut into 3 μm thick sections with Leica Microtome. The renal sections were stained with periodic acid-schiff (PAS) and Hematoxylin & Eosin (HE), and liver sections with oil red O reagent, separately. The morphological changes of glomerulus were observed with KF-PRO-005-EX digital scanner (Motic, Xiamen, China).

The images were selected randomly in which the glomerulus number was no more than twenty and were analyzed with K-Viewer (1.5.3.1) image analysis software (×400, KFMI, China). The length of the two longest perpendicular diameters in each glomerular capillary tuft without Bowman’s space was measured in μm, and then the mean value was calculated from 10 images. The areas of the glomerular mesangial region and capillary tuft were measured. The relative area of the mesangial region (%) was calculated according to the formula (Zhang et al., 2022).

$$\text{The}\;\text{relative}\;\text{area}\;\left(\%\right)=\frac{\text{Area}\;\text{of}\;\text{the}\;\text{mesangial}\;\text{region}}{\text{Area}\;\text{of}\;\text{the}\;\text{capillary}\;\text{tuft}}\times 100$$

#### 2.9.2 Electron microscopy

Freshly dissected kidneys were fixed using 2.5% glutaraldehyde. The ultrathin sections were stained with uranium acetate-lead citrate for electron microscopy. For each specimen, ten photographs (×20,000 magnification) covering different regions in the glomerular cross section were taken separately.

The thickness of glomerular basement membrane (GBM) and the width of glomerular foot process (GFP) were all measured. Images were selected and analyzed using the RADIUS Control & Imaging software (EMSIS ASIA, Germany).

### 2.10 Western blot

Renal tissues (about 20 mg) were homogenized with lysis buffer (1:10, w/v) containing protease inhibitors. After centrifugation at 12,000 rpm for 10 min at 4°C, the supernatants were collected and protein levels were determined using the BCA protein assay kit (Epizyme Biotech, Shanghai, China). Proteins were separated by 10% SDS-PAGE and then transferred to a PVDF membrane. Membranes were blocked with protein free rapid blocking buffer (Epizyme Biotech, Shanghai, China) for 20 min and probed with primary antibodies overnight at 4°C, including Nephrin (Abcam, United States) and Synaptopodin (Proteintech, China). GAPDH (Abways, China) was used as loading control. After the membrane was washed with TBST three times, it was incubated with HRP-conjugated anti-rabbit antibody for 60 min at room temperature. The bands were visualized by Tanon (Shanghai, China) after using a chemiluminescence reagent.

### 2.11 Immunohistochemistry

Renal tissues were fixed in 10% buffered formalin for 3 days, embedded in paraffin and then sectioned at a thickness of 4 mm. After deparaffinization, antigen retrieval and blocking with goat serum, the section was incubating with anti-IL-6 or anti-TNF-α rabbit polyclonal antibodies (Servicebio, Wuhan, China) overnight at 4°C. After washed with PBS, the sections were incubated with HRP-conjugated goat anti-rabbit IgG secondary antibody (Servicebio, Wuhan, China) and the peroxidase reaction was developed with diaminobenzidine (Servicebio, Wuhan, China). Subsequently, the sections were counterstained with hematoxylin, and the images were captured using an inverted microscope.

### 2.12 Animal experiments of IO components absorbed into blood

Three 8-week-old C57BL/6J male mice, weighing (24.6 ± 1.0) g, were acclimatized and fed for 1 week, and then the mice were administered with IO at 300 mg/kg by gavage, the maximum dose available, twice a day for 3 days. The mice were anesthetized and the blood was taken from the eye socket within 1 hour of the last administration. The three blood samples were centrifuged separately for 10 min at 4°C, 3,000 rpm, and then the supernatants were mixed well as drug-containing serum.

The 400 μL drug-containing serum was add with 600 μL of methanol and centrifuged at 17,000 g for 15 min at 4°C. The supernatant was taken and dried under vacuum. The dried sample was dissolved in 100 μL methanol (50%, in water). After vortexing, the supernatant was analyzed by an UPLC system (Shimadzu, JPN). Chromatographic separation and mass spectrometric methods were performed as in 2.2.

### 2.13 Molecular docking

The 3D structure information of IO against ORG compounds we obtained in PubChem. Then, the 3D structure information of the target protein (TNF-α and IL-6) was obtained in PDB database. The structures of large molecule target proteins and small molecule compounds were imported in CB-DOCK2 website (https://cadd.labshare.cn/cb-dock2/php/index.php) (Liu Y. et al., 2022) for virtual molecular docking.

### 2.14 Statistical analysis

Measurement data were expressed as mean ± standard deviation ( $\bar{X}$ ± SD). The comparison of sample means between the two groups was performed using the independent samples *t*-test. The difference between the two groups was considered statistically significant at *p* < 0.05.

## 3 Results

### 3.1 Construction of PPI network

**FIGURE 1:**
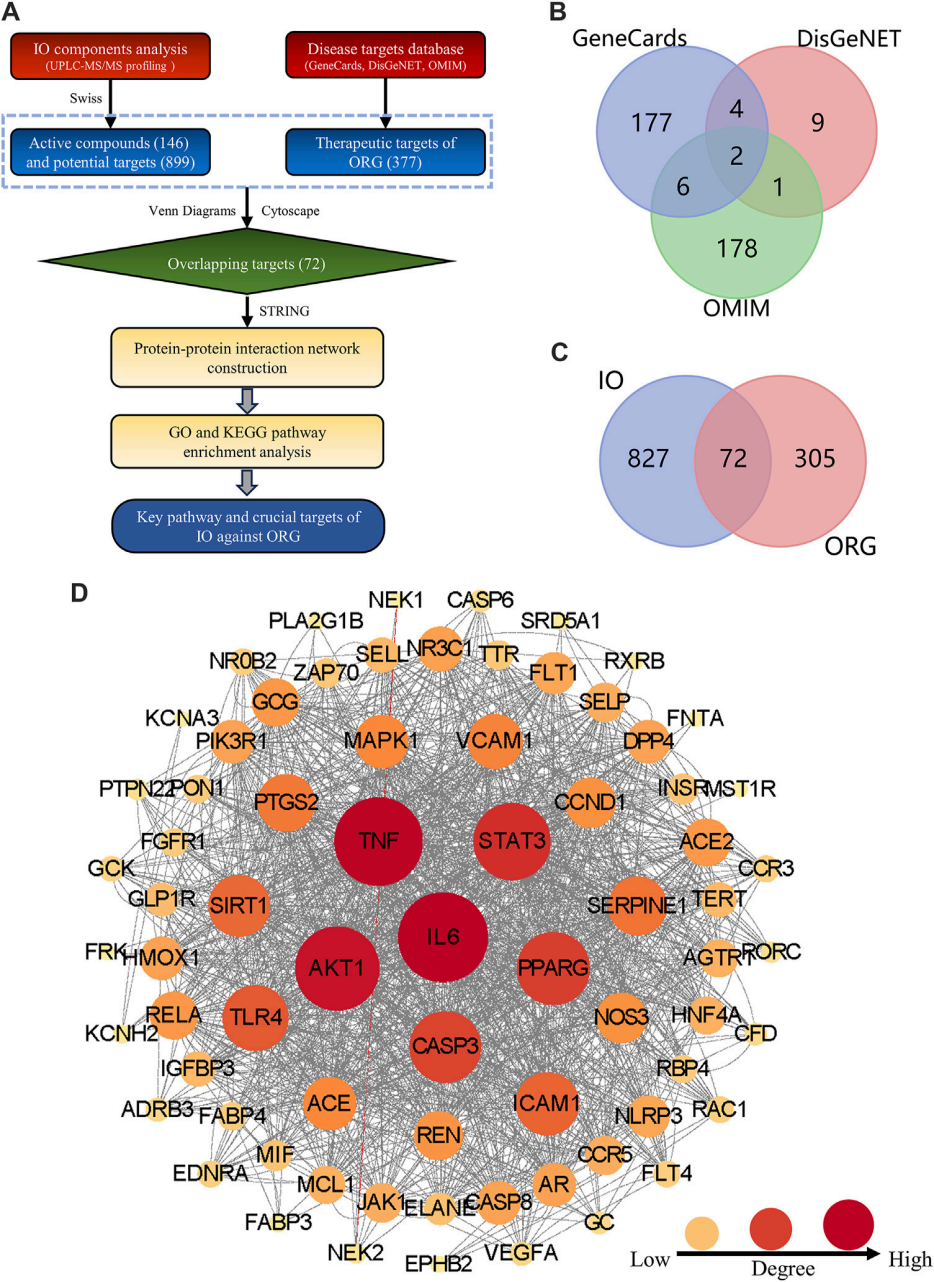
Construction the PPI network of key targets for IO against ORG. **(A)** Flowchart of network pharmacology. **(B)** ORG targets based on GeneCards, DisGeNet and OMIM databases. **(C)** Overlapping targets between IO and ORG. **(D)** PPI analysis of IO against ORG. IO: *Inonotus obliquus*; ORG: obesity-related glomerulopathy; GO: Gene ontology; KEGG: Kyoto Encyclopedia of Genes and Genomes; PPI: protein-protein interaction.

The flowchart of network pharmacology and UPLC-Q-TOF-MS integrated strategy, to reveal the mechanism of IO against ORG, was shown in Figure 1A. A total of 146 components were identified in the IO using UPLC-Q-TOF-MS analysis (Supplementary Table S1). The total ion flow diagram of IO extracts was presented in Supplementary Figure S1.

The structural formulae of the 146 components obtained from UPLC-Q-TOF-MS profiling analysis were imported into the SwissTargetPrediction database for the prediction of targets. There are 5,378 possible potential targets corresponding to 115 components, but no potential targets for the other 31 components. After de-duplication and harmonization of Uniprot IDs, 899 potential targets were finally obtained.

By searching in the disease-target platform, 189, 16, and 187 therapeutic targets for ORG were collected from GeneCards, DisGeNET and OMIM databases, respectively. Then, the results of the three databases were merged, and a total of 377 ORG therapeutic targets were obtained (Figure 1B). Only 72 key targets from the intersection of 899 IO potential targets with 377 ORG therapeutic targets were kept (Figure 1C).

The PPI network of 72 key targets was constructed, which had 72 nodes and 1236 edges with an average degree of 34.3. Among them, 31 core targets, such as IL-6, TNF, AKT1, STAT3, and PPARG, were greater than the average degree value (Supplementary Table S2). The results suggested that core targets, such as IL-6 and TNF, played key regulatory roles in the PPI network of 72 targets for IO against ORG (Figure 1D).

### 3.2 Enrichment analysis of the key regulation pathway for IO against ORG

**FIGURE 2:**
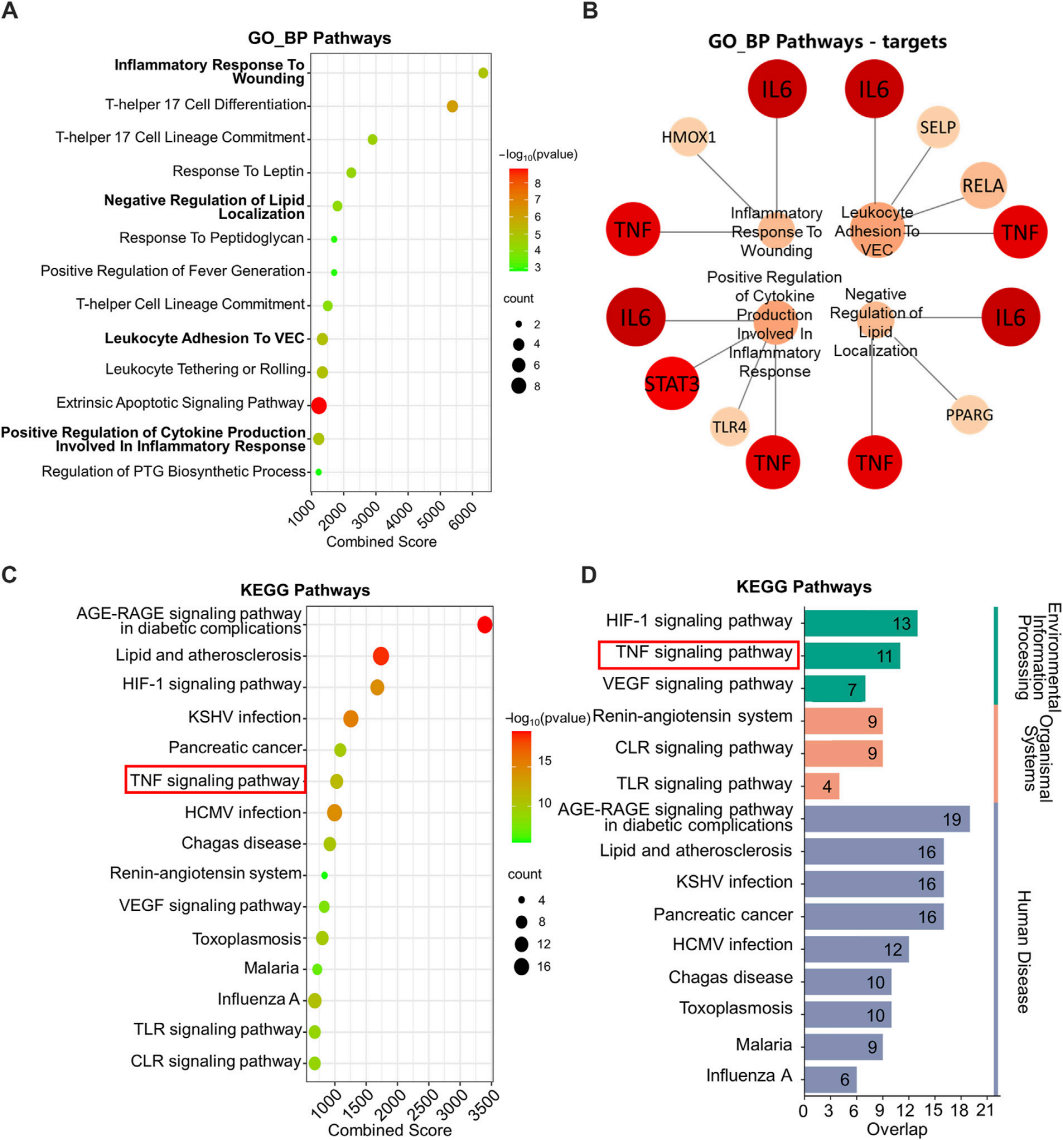
GO_BP and KEGG enrichment analysis. **(A)** GO_BP enrichment analysis. **(B)** Network plot of GO_BP pathways involving TNF and IL6. **(C)** KEGG enrichment analysis. **(D)** Classified KEGG pathways. GO_BP: Gene ontology biological process; KEGG: Kyoto Encyclopedia of Genes and Genomes. The following are database name abbreviations: VEC: vascular endothelial cell; PTG: prostaglandin; KSHV: kaposi sarcoma-associated herpesvirus; HCMV: human cytomegalovirus; VEGF: vascular endothelial growth factor; TLR: toll-like receptor; CLR: C-type lectin receptor.

KEGG and GO_BP pathway enrichment analysis was performed on the 72 key targets. In Figure 2A, GO_BP pathway enrichment analysis revealed that pathways regulating inflammatory response were significantly enriched, e.g., inflammatory response to wounding and positive regulation of cytokine production involved in inflammatory response.

Moreover, by performing targets analysis on the enriched pathways, we found that TNF and IL-6 involved in four pathways associated with inflammation. They were inflammatory response to wounding, negative regulation of lipid localization, positive regulation of leukocyte adhesion to vascular endothelial cell and positive regulation of cytokine production involved in inflammatory response (Figure 2B).

The KEGG pathway enrichment analysis suggested that the treatment mechanism of IO for ORG might be related to TNF signaling pathway, HIF-1 signaling pathway and the pathways of glycolipid metabolism, such as AGE-RAGE signaling pathway in diabetic complications and lipid and atherosclerosis (Figure 2C).

Further categorization of the pathways with top 15 combined scores showed that HIF-1 and TNF signaling pathways were key regulatory pathways in the environmental information processing classification (Figure 2D). Combined with the results of PPI, the mechanism may be related to the regulation of TNF and IL-6 in the TNF signaling pathway.

### 3.3 Improvement of IO on obesity-related biological parameters

#### 3.3.1 Improvement of IO on obesity

**FIGURE 3:**
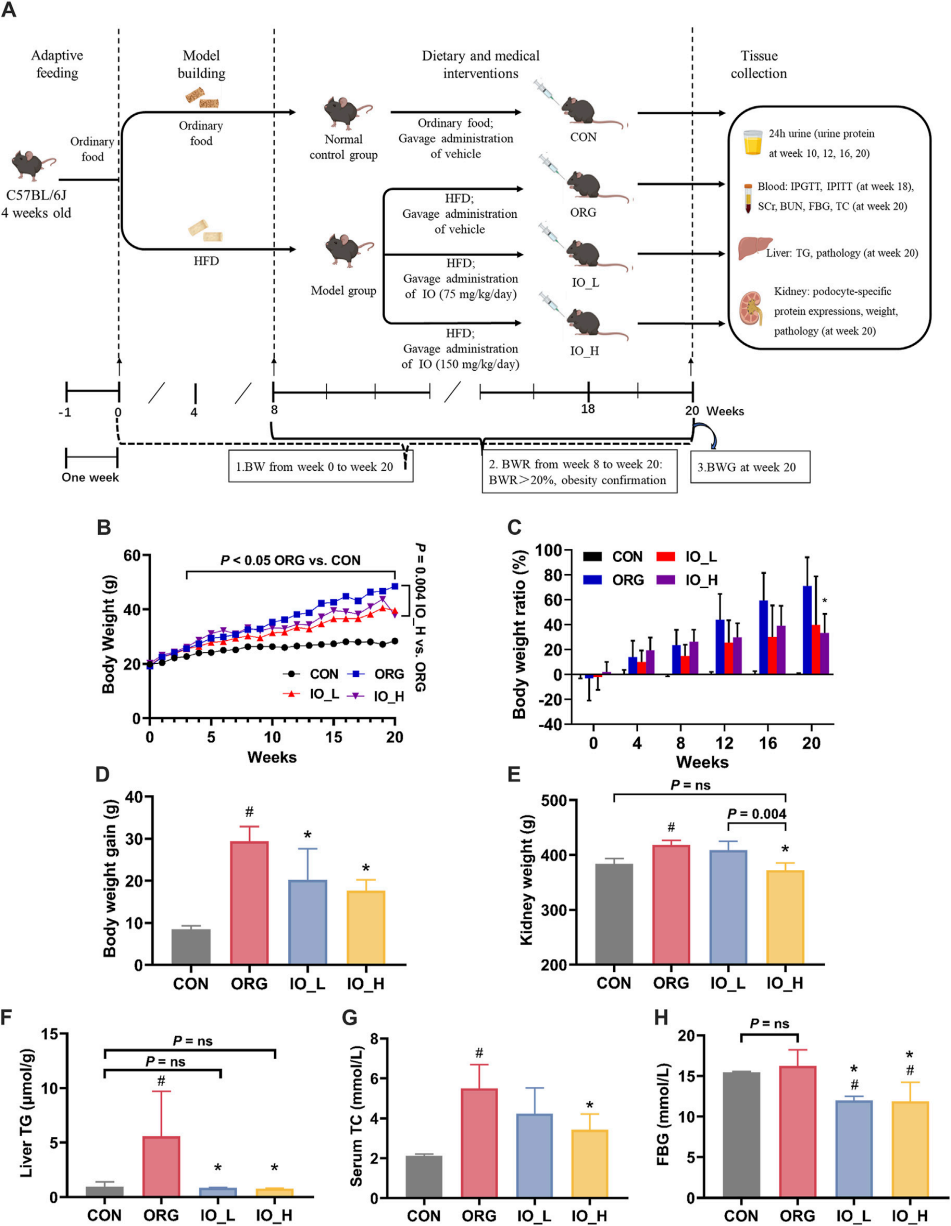
Effects of IO on HFD-induced obesity and obesity-related biological parameters. **(A)** Flowchart of animal experiments. **(B)** Body weight. *p* < 0.05: ORG vs. CON from week 4 to week 20. **(C)** Body weight ratio. **(D)** Body weight gain. **(E)** Kidney weight. **(F)** Levels of liver TG. **(G)** Levels of serum TC. **(H)** Levels of FBG. HFD: high-fat-diet; CON: the control group; ORG: the obesity-related glomerulopathy model; IO_L: the model group treated with *Inonotus obliquus* (75 mg/kg/day); IO_H: the model group treated with *I. obliquus* (150 mg/kg/day); BW: body weight; BWR: body weight ratio; BWG: body weight gain; TG: triglyceride; TC: total cholesterol; SCr: serum creatinine; BUN: blood urea nitrogen; FBG: fasting blood glucose. Data were presented as mean ± SD. ^#^ *p* < 0.05 vs. the CON; ^*^ *p* < 0.05 vs. the ORG.

The flowchart of animal experiments was shown in Figure 3A. From week 0 to week 20, the results of BW of the different groups were shown in Figure 3B. From week 4 to the end of the experiment, the BW of the model group, measured weekly, increased significantly compared to that of the CON group (all: *p* < 0.05).

At week 8, the model group gained 20% more BW than the CON group (BWR >20%), at which time point the obesity modeling was considered to be established (Wang Y. N. et al., 2023). As shown in Figure 3C, from week 8 to week 20, the BWR values between the model and CON groups were all higher than 20% and gradually increased with feeding time. At week 20, the BWR value in the model group increased to 79.9%, which was significantly higher than the BWR value in the IO_H group (*p* < 0.01).

Body weight gain (BWG) is another calculated indicator to assess the obesity of the same experimental group with feeding time (Han et al., 2023). The BWG of each experimental group was calculated as follows.

$$\text{The}\;\text{BWG}\;\left(g\right)=\left(\text{The}\;\text{average}\;\text{BW}\;\text{at}\;\text{week}\;20\right)-\left(\text{The}\;\text{average}\;\text{BW}\;\text{at}\;\text{week}\;0\right)$$

We could see in Figure 3D that the BWG from week 0 to week 20 of the ORG group was significantly higher than the CON group (*p* < 0.001). After IO intervention, the BWG of the mice in the IO_L and IO_H group was significantly reduced when compared to the ORG group (IO_L: *p* = 0.029; IO_H: *p* < 0.001).

#### 3.3.2 Improvement of IO on kidney weight

The results of kidney weight were shown in Figure 3E. The kidney weight was significantly increased in the ORG group compared with the CON group (*p* < 0.001). After IO intervention, the IO_H group was not only significantly reduced compared with the ORG group (*p* < 0.001) and the IO_L group (*p* = 0.004) separately, but also not statistically different from the CON group (*P* = ns).

#### 3.3.3 Improvement of IO on hepatic TG and serum TC

As shown in Figure 3F, the ORG mice had the highest hepatic TG content, and the differences were statistically significant compared to the CON group (*p* = 0.040), IO_L administration (*p* = 0.037) and IO_H administration (*p* = 0.035). The CON group, IO_L group and IO_H did not show any significant difference (Three: *P* = ns). The results of oil red O staining on liver tissue also had the same trend (Supplementary Figure S2).

Serum TC level is a commonly used clinical index for the diagnosis of hyperlipidemia (Yin et al., 2023). As shown in Figure 3G, serum TC was significantly elevated in the ORG group compared to the CON group (*p* < 0.001). Serum TC in the IO_H group decreased statistically compared with the ORG group (*p* = 0.005).

#### 3.3.4 Improvement of IO on FBG, IPGTT, and IPITT

At 20th week, the FBG levels of the mice in each group were shown in Figure 3H. FBG level was elevated in the ORG group after HFD feeding but was not significantly different from the CON group (*P* = ns), which is consistent with the clinical diagnosis of ORG (Yang et al., 2024).

Notably, FBG levels were significantly lower in both the IO_L and IO_H groups when they compared to the ORG group and the CON group separately (four: *p* < 0.05), but the lowered FBG still remained within the normal range.

As shown in Supplementary Figure S3, the results of IPGTT and IPITT assay also indicated the beneficial effect of IO on glucose metabolism in the model group.

### 3.4 Improvement of IO on renal function and pathological examination

#### 3.4.1 Improvement of IO on BUN, SCr and urinary protein

The results of BUN and SCr were shown in Supplementary Figure S4. There was no significant difference in BUN and SCr between the CON and ORG group. The trends of the IO_H group and the IO_L group on BUN and SCr level were similar as FBG level.

**FIGURE 4:**
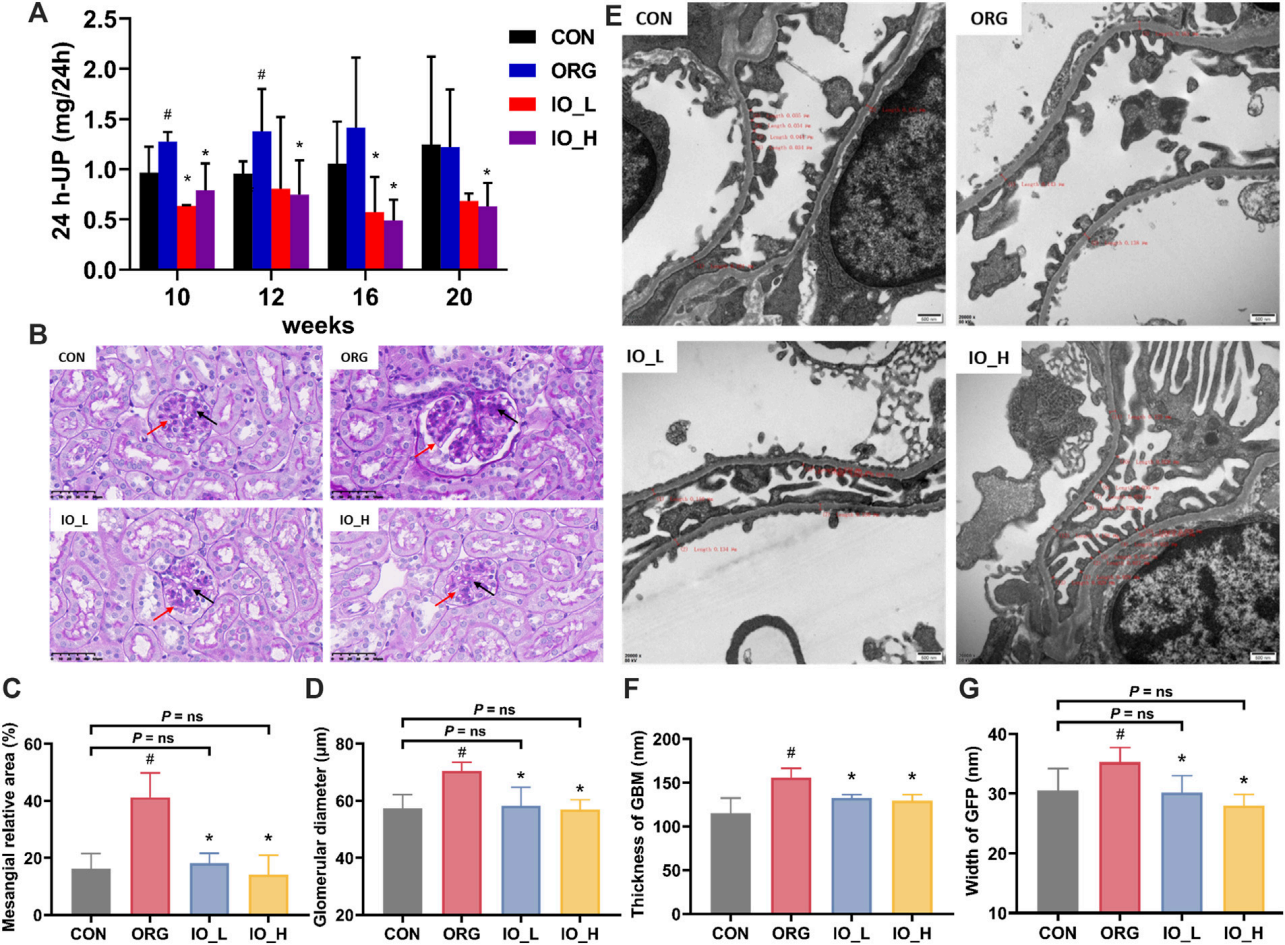
Effects of IO on urinary protein and renal pathology in ORG mice. **(A)** Urinary protein excretion during 24 h. **(B)** Light microscopic images of glomerulus (PAS staining, × 400). **(C)** Mesangial relative area. **(D)** Glomerular diameter. **(E)** Electron microscopic images of podocyte GBM and GFP (×20,000). **(F)** Thickness of GBM. **(G)** Width of GFP. Red arrow: glomerular basement membrane; Black arrow: glomerular mesangial matrix. GBM: glomerular basement membrane; GFP: glomerular foot process; CON: the control group; ORG: the obesity-related glomerulopathy model; IO_L: the model group treated with *Inonotus obliquus* (75 mg/kg/day); IO_H: the model group treated with *I. obliquus* (150 mg/kg/day). Data were presented as mean ± SD. ^#^ *p* < 0.05 vs. the CON; ^*^ *p* < 0.05 vs. the ORG.

The results of 24-h urine protein of mice in each group were shown in Figure 4A. The 24-h urine protein of the ORG group significantly increased at week 10 and week 12 when compared to the CON group at same testing time (*p* < 0.05), however, at week 16 and week 20, there was no significant difference between the above two groups. At the four monitoring points from week 10 to week 20, 24-h urinary protein was significantly reduced in the IO_H group compared to the ORG group (*p* < 0.05).

#### 3.4.2 Improvement of IO on pathological examination

As shown in Figure 4B, PAS staining showed glomerular hypertrophy in the ORG group, which was improved by the administration of IO.

Figures 4C,D showed the results of mesangial relative area and glomerular diameter of the four groups. Compared with the CON group, the ORG group had significantly increased mesangial relative area and glomerular diameter (two: *p* < 0.001). After 12 weeks of IO intervention, the glomerular diameter and the mesangial relative area were significantly improved compared with those in the ORG group (all: *p* < 0.001) and had no significant difference with the CON group (all: *P* = ns).

The results of HE staining also revealed the pathological changes in the glomerulus of the mice in each group, which were consistent with those of PAS staining (Supplementary Figure S5).

As shown in Figure 4E, the foot process in the CON group were arranged in an orderly manner, uniform in size. While in the ORG group, fusion of foot process could be observed, and it was also accompanied by apoptosis of podocytes. In the IO_L and IO_H groups, significant improvement could be seen.

In Figures 4F,G, the thickness of GBM and the width of GFP were significantly increased in the ORG group compared with the CON group (*p* < 0.05). The thickness of GBM and the width of GFP were significantly improved in the IO_L and IO_H groups compared with the ORG group (all: *p* < 0.001). Figure 4G also showed that the GFP widths of IO_L and IO_H were not significantly different from the CON group (*P* = ns).

### 3.5 Improvement of IO on podocyte specific proteins in ORG mice

**FIGURE 5:**
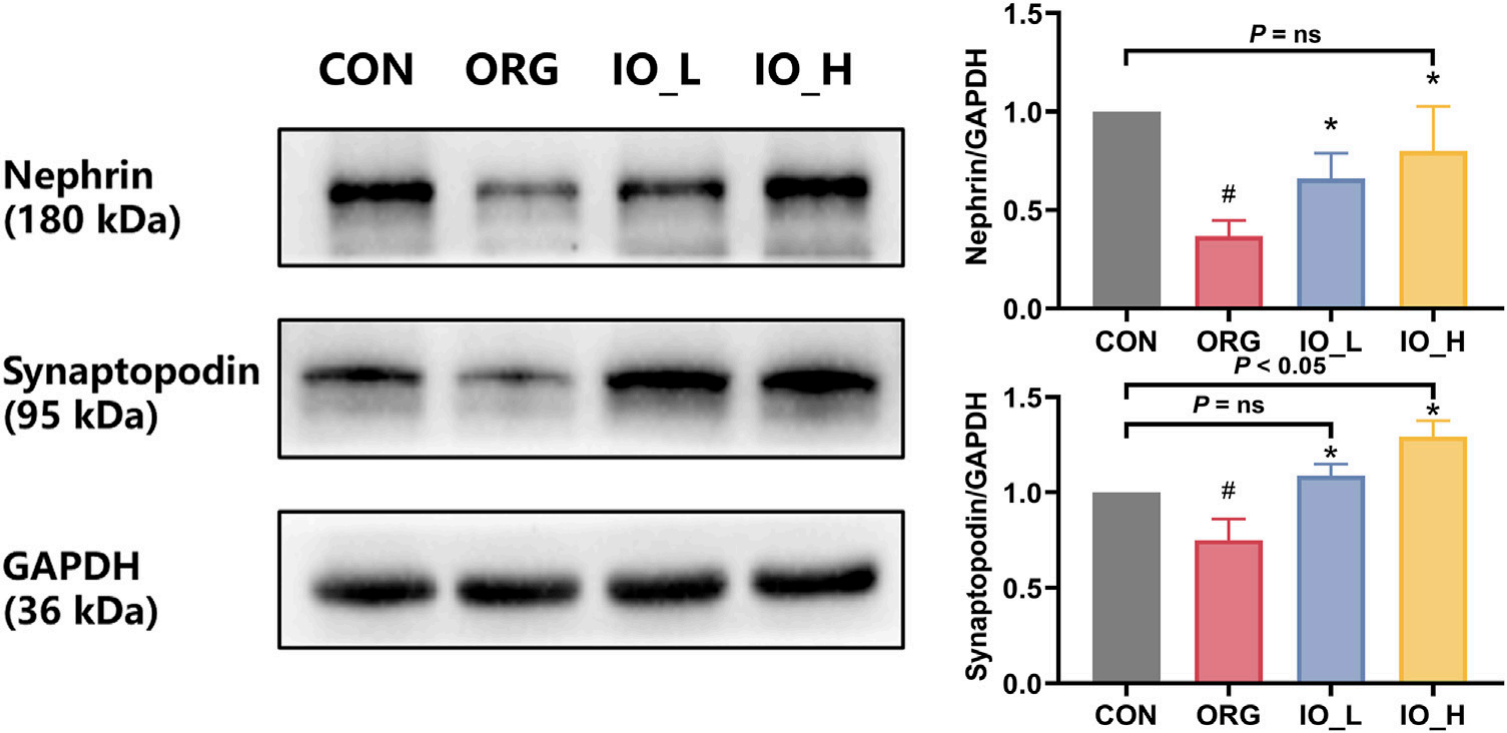
The protein expressions of renal nephrin and synaptopodin. CON: the control group; ORG: the obesity-related glomerulopathy group; IO_L: the model group treated with *Inonotus obliquus* (75 mg/kg/day); IO_H: the model group treated with *I. obliquus* (150 mg/kg/day). Data were presented as mean ± SD. ^#^ *p* < 0.05 vs. the CON; ^*^ *p* < 0.05 vs. the ORG.

Figure 5 showed that the protein expressions of nephrin and synaptopodin were significantly decreased in the ORG group compared to the CON group (*p* < 0.05). After 12 weeks of IO intervention, the two podocyte specific protein expressions in IO_L and IO_H group were significantly increased when compared to the ORG group (*p* < 0.05).

Also of interest was that after IO intervention in the high dose, nephrin did not differ from the CON group (*P* = ns), whereas synaptopodin was significantly better than the CON (*p* = 0.027).

### 3.6 Regulation of IO on TNF-α and IL-6 levels in ORG mice

**FIGURE 6:**
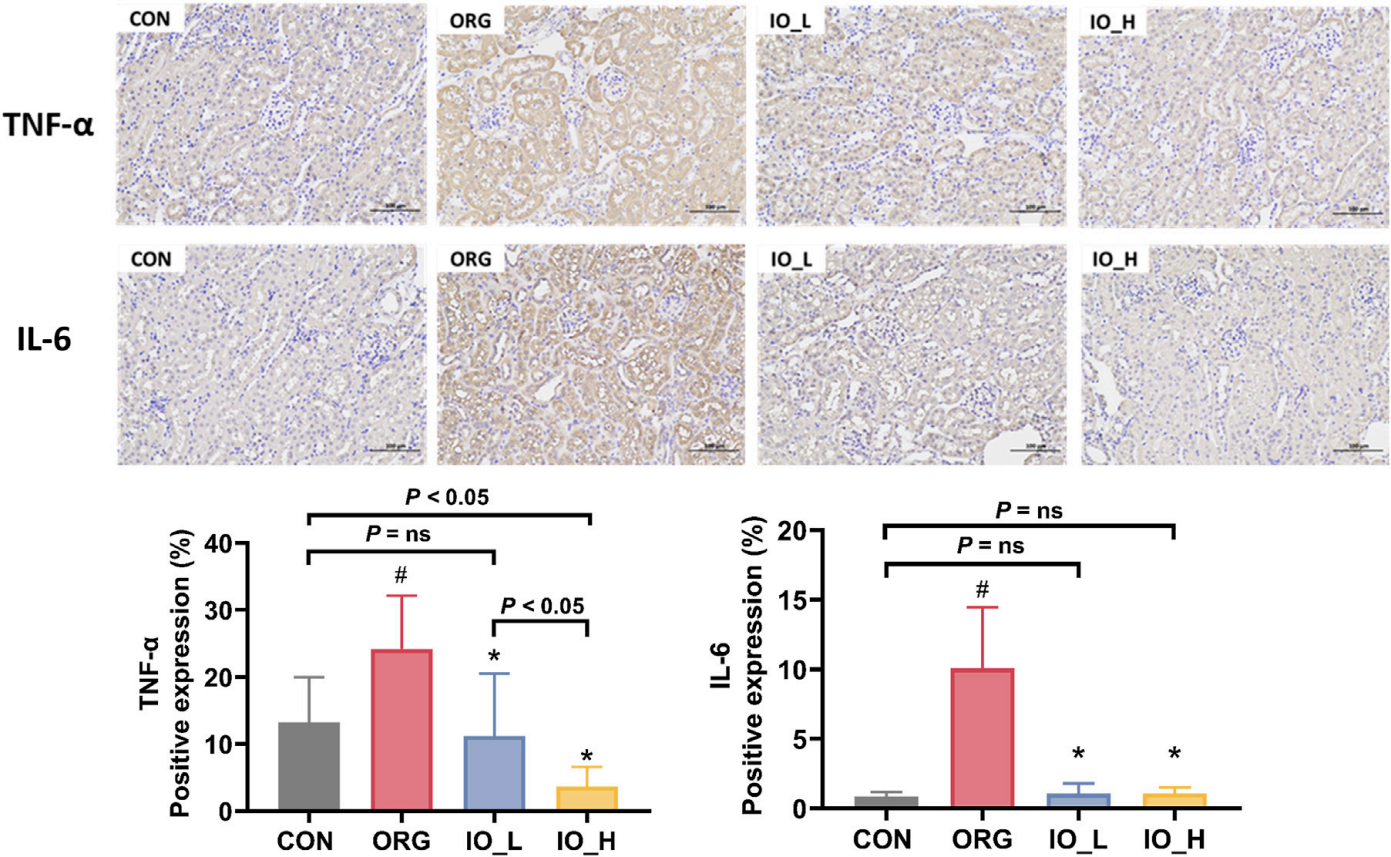
The expressions of renal TNF-α and IL-6 (×200). CON: the control group; ORG: the obesity-related glomerulopathy model; IO_L: the model group treated with *Inonotus obliquus* (75 mg/kg/day); IO_H: the model group treated with *I*. *obliquus* (150 mg/kg/day). Data were presented as mean ± SD. ^#^ *p* < 0.05 vs. the CON; ^*^ *p* < 0.05 vs. the ORG.

The immunohistochemical staining results of TNF-α and IL-6 in the kidney tissue were shown in Figure 6. We could see that the expression levels of TNF-α and IL-6 were significantly higher in the ORG group compared with the CON group (*p* < 0.05).

After 12 weeks intervention with IO, both TNF-α and IL-6 level in the two IO groups improved significantly compared to the model (*p* < 0.05), but only TNF-α level showed a dose-dependent manner (*p* < 0.05).

### 3.7 The compounds of IO absorbed into blood for ORG treatment

**FIGURE 7:**
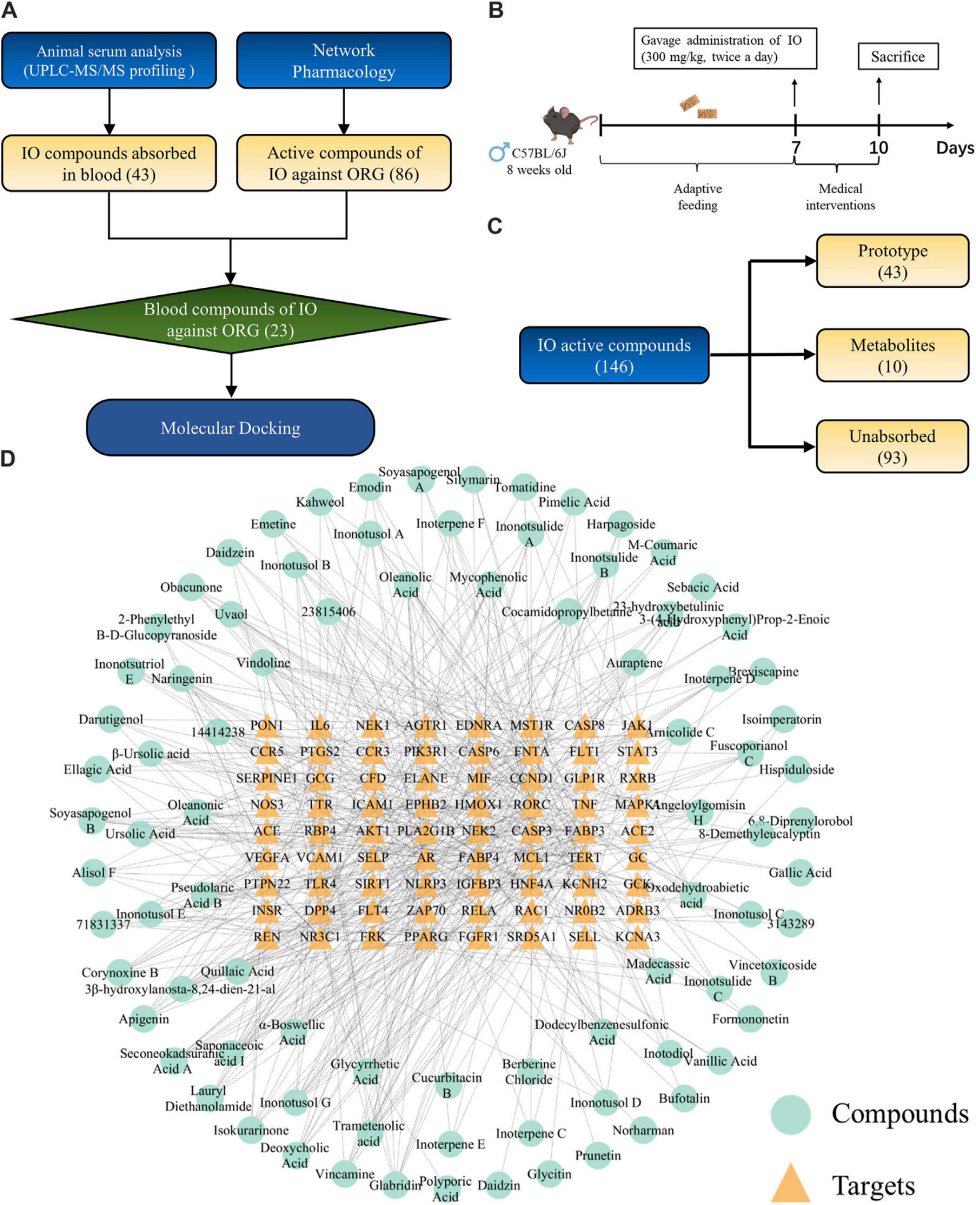
Exploration of active compounds in IO against ORG mice. **(A)** Flowchart of the exploration of active compounds in IO against ORG mice. **(B)** Study of the component analysis in mouse blood after IO administration. **(C)** The result of **(B)**. **(D)** “compounds-targets” network of IO against ORG. IO: *Inonotus obliquus*; ORG: obesity-related glomerulopathy.

The flowchart of network pharmacology and UPLC-Q-TOF-MS integrated strategy to explore the active compounds of IO against ORG was shown in Figure 7A. The preparation of drug-containing serum in mice was shown in Figure 7B. UPLC-Q-TOF-MS analysis showed that there were 146 compounds in the IO extracts, of which 43 compounds were absorbed as prototypes and 10 compounds as their own metabolites (Figure 7C; Supplementary Table S3). The mass spectrometry spectrum was shown in Supplementary Figure S6.

Small molecules typically exert their bioactive effects through interactions with protein targets. In order to identify the interaction between the 72 key targets of IO against ORG and IO compounds, a “compounds-targets network” was established in Figure 7D.

**FIGURE 8:**
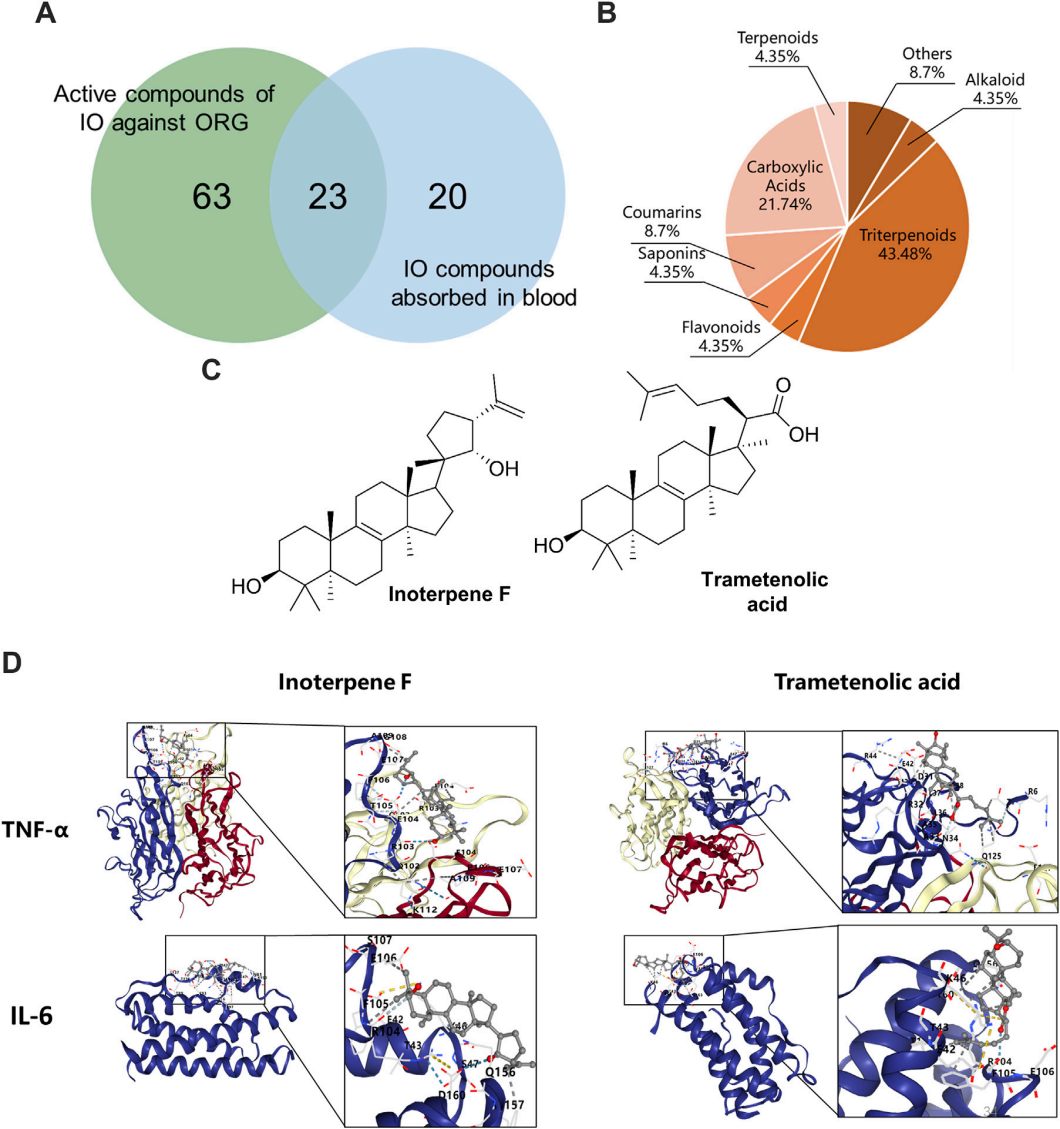
Molecular docking of IO against ORG based on active compounds and targets. **(A)** The overlapping compounds between IO against ORG and IO absorbed in blood. **(B)** Classification of active compounds of IO against ORG. **(C)** Structural formula of interpene F and trametenolic acid. **(D)** Molecular docking of interpene F and trametenolic acid with TNF-α and IL-6. IO: *Inonotus obliquus*; ORG: obesity-related glomerulopathy.

The compounds-targets network contained 72 key targets and 86 potential active compounds, and 513 compound-target interactions were generated. As shown in Figure 8A, only 23 common compounds from the intersection of 45 absorbed compounds of the IO extracts with 86 potential compounds from network analysis were kept. Among the 23 active compounds, there were 10 triterpenoid components, which accounted for the largest share of 43.48% (Figure 8B). These results indicated that triterpenoid components, such as inoterpene F and trametenolic acid, were active compounds in the regulation of TNF-α and IL-6.

### 3.8 Validation of triterpenoids on TNF signaling pathway

The binding energy of molecular docking indicates the energy value of receptor-ligand docking, and the smaller the value, the greater the affinity of the ligand-receptor binding and the more stable the conformation (Figure 8C). Inoterpene F and trametenolic acid are two kinds of triterpenoid monomers extracted from IO by our research group (Hao, 2023). The binding energy of inoterpene F was −7.1 kcal/mol with TNF-α and −6.9 kcal/mol with IL-6; the binding energy of trametenolic acid was −6.9 kcal/mol with TNF-α and −7.0 kcal/mol with IL-6 (Figure 8D). These results confirmed that the triterpenoid components of IO extracts could directly bind to TNF-α and IL-6, to regulate the inflammatory signaling pathway, and improve the ORG.

### 3.9 Prediction of the active components based on network pharmacology

**FIGURE 9:**
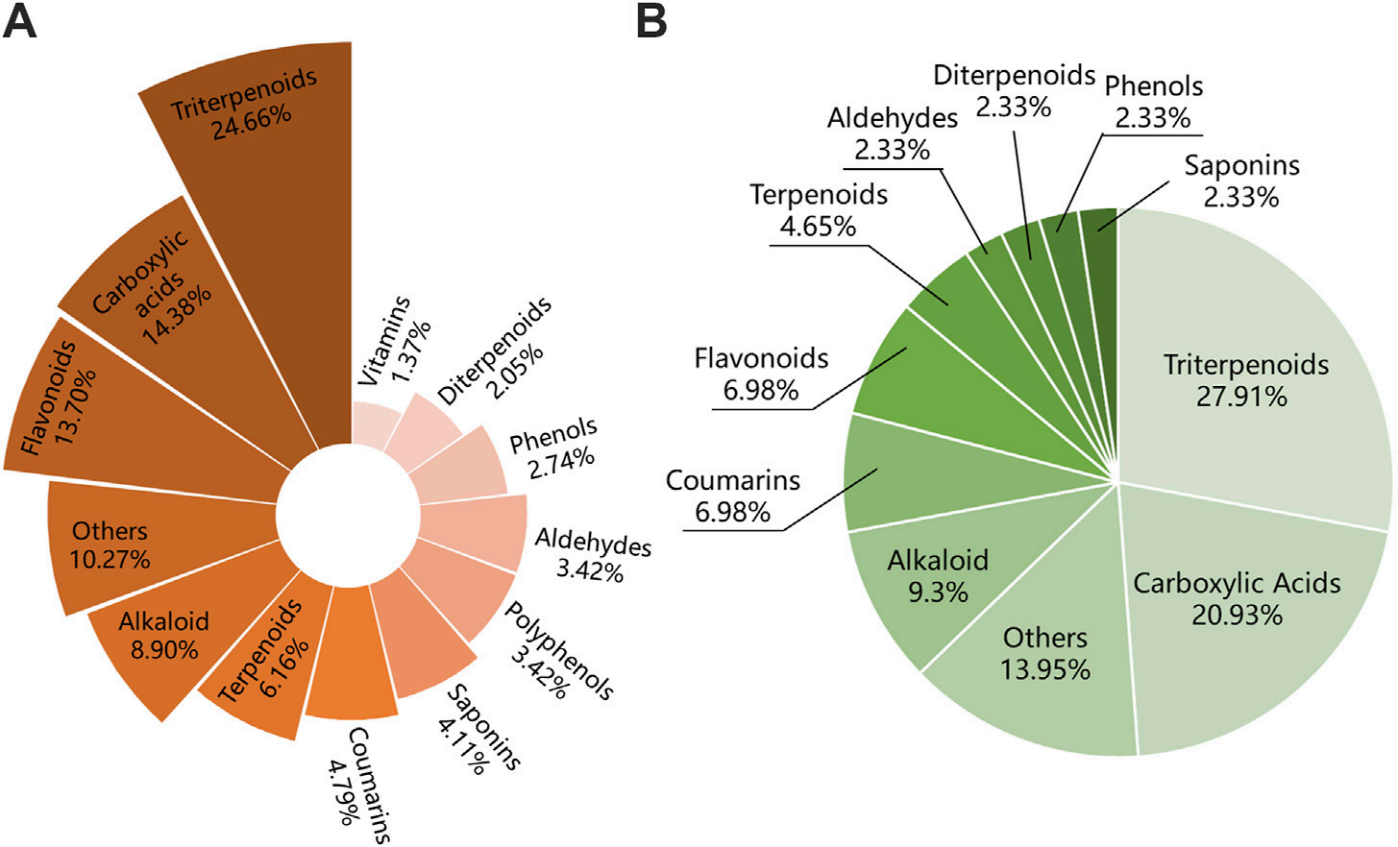
Compounds classification of IO extracts and those which were absorbed into bloodstream. **(A)** Compounds classification of IO extracts. **(B)** Compounds classification of IO extracts which were absorbed into bloodstream. IO: *Inonotus obliquus*.

Among the 146 components of the IO extracts, there were 48 terpenoids, including 36 triterpenoids, accounting for about 24.66% (Figure 9A). Among the 43 components of IO absorbed into blood, there were 12 triterpenoids, accounting for 27.91% (Figure 9B). Combining the results of network analysis and molecular docking, it is obvious that the triterpenoid compounds of IO was not only the main compounds but also the active components.

## 4 Discussion

Currently, obesity is increasing worldwide at an alarming rate. Obesity increases the risk of many chronic diseases, such as type 2 diabetes, coronary heart disease, and also ORG. The prevalence of ORG is increasing in parallel with the worldwide obesity epidemic. The body mass index and waist circumference are used a lot to classify obesity in clinical medicine (Nimptsch et al., 2019). The obesity mice model could be verified based on BW (Ren et al., 2022), BWG (Han et al., 2023) and BWR (Wang Y. et al., 2023) which were all observed in our study. Rodent models have been widely used to elucidate the pathophysiology of obesity with success for decades, but how to define obesity in rat or mouse rodent models remains ambiguous, just like lacking a globally accepted definition of obesity in human (Vecchié et al., 2018). In this case, three indicators, BW, BWR and BWG, were used to determine the degree of obesity in a comprehensive manner.

After 4 weeks of HFD feeding, the BW between the model and the normal showed a significant difference, which lasted until 8 weeks when the BWR rose to 20% (Wang et al., 2023a) and the obesity model was confirmed. The results of the next 12 weeks of intervention research, in terms of BW, BWR and BWG, confirmed the weight loss effect of IO on obese mice. This is not the first report that IO has an effect on weight loss in an animal model of obesity (Yu et al., 2020; Yu et al., 2021; Hu et al., 2024), but it might be the first study of IO on weight loss and renal protection in ORG mice.

The pathogenesis of ORG is mainly related to glomerular hemodynamic changes and lipid ectopic deposition caused by obesity (Wei et al., 2021). In clinical practice, ORG is mainly characterized by asymptomatic proteinuria and its prominent pathological change is obvious glomerular hypertrophy (Wang and Baozhang, 2022).

The pathogenesis of ORG is mainly related to glomerular hemodynamic changes and lipid ectopic deposition caused by obesity (Wei et al., 2021). In clinical practice, ORG is mainly characterized by asymptomatic proteinuria and its prominent pathological change is obvious glomerular hypertrophy (Wang and Baozhang, 2022). In our study, the glomerular hypertrophy and the expression of podocyte specific proteins indicated podocyte injury but there were no significant changes in 24-h urinary protein. Pathological studies have confirmed that less than 30% podocyte damage does not cause glomerulosclerosis, which associated with the severity of CKD (Bhargava et al., 2021). This may explain why our model has not yet developed proteinuria despite podocyte injury. Proteinuria may be observed with prolonged HFD or improved assays of 24-h urinary protein. According to clinical practice, the mice podocyte injury model established in this study is regarded as an early ORG disease model (Song et al., 2023).

In addition, the apparent effects of IO in improving basement membrane thickness, fusion and disappearance of podocyte foot processes (Gong et al., 2023) as well as podocyte-specific protein expressions in model mice suggested that IO possessed the cell biology as well as molecular biological potential to improve proteinuria in ORG. This potential was confirmed in the urine sample assay.

The relationship between increased expression of nephrin and podocalyxin and reduced proteinuria was confirmed in some pharmacologic intervention studies (Wang Y. N. et al., 2023). Our previous study has shown that nephrin belongs to the slit diaphragm proteins and podocalyxin belongs to the apical membrane protein (Gong et al., 2023). We will continue to investigate the effects of IO on other podocyte-specific proteins such as cytoskeletal proteins (e.g., synaptophysin) and basement membrane proteins (e.g., integrin α3β1) in order to compensate for the shortcomings of the present experiments.

Injury-induced changes in podocyte physiology and function are a complex interaction of multiple proteins (Gong et al., 2023) that comprise the anatomical structure of podocytes at molecular levels (Lu et al., 2019). The clinical symptom of podocyte injury is proteinuria, sometimes with loss of kidney functions progressing to glomerulosclerosis (Lu et al., 2019). Our previous study showed that the presence of proteinuria in animal model is directly related to podocyte injury (Feng et al., 2024). This study suggested that podocyte injury occurred at the molecular level and had not progressed to proteinuria yet.

In the present study, we also observed that IO reduced obesity-induced elevated serum TC, liver TG and FBG. In clinical diagnosis, normalization of blood glucose is one of the indicators to differentiate between ORG and DKD (Scurt et al., 2024). In our study, obesity caused significant elevation of TC and TG in the model group without significant change in blood glucose, but IO still showed a blood glucose lowering effect. The above study showed the overall modulatory effect of IO on disorders of glycolipid metabolism.

As shown by literature data, various authors have tried to determine the molecular mechanism of action of IO extracts (Szychowski et al., 2020). In our study, mechanistic investigations revealed that the regulation of IO against ORG was closely related to TNF-α and IL-6 in the TNF signaling pathway. Several studies have recently highlighted the critical role of inflammation in ORG development. The relationship between obesity and ORG is facilitated by a network of various inflammation-associated cells (e.g., synaptophysin) and a series of inflammatory mediators (e.g., TNF-α and IL-6) (Guan et al., 2024). TNF-α and IL-6 are both small molecule proteins secreted by monocyte macrophages, which can induce apoptosis or inflammatory responses by activating different signaling pathways (Guo et al., 2019; Sethi and Hotamisligil, 2021). Our research indicated that the renoprotective effects of IO was related with it suppression on TNF-α and IL-6 levels.

On the principle that the renoprotective effects and related-mechanism of IO had been demonstrated, our study on basis of the pharmacokinetics explored the components of IO extracts that are absorbed into the blood and had therapeutic effects on ORG. It has been shown that triterpenoids of IO could maintain the homeostasis of glycolipid metabolism (Tian et al., 2022). *In vitro* experiments showed that triterpenoids of IO had hypoglycemic effects by inhibiting α-glucosidasein (Chen et al., 2021). In addition, triterpenoids of IO could reduce the viscosity of platelets, making them more difficult to build up in the arterial wall, but also can remove free radicals in the body, play an anti-inflammatory role (Duru et al., 2019; Kou et al., 2022). In particularly, trametenolic acid was described to ameliorates the progression of DKD in db/db mice via inflammation pathways (Duan et al., 2022).

When lipid metabolism is disturbed, such as in hyperlipidemic states, excess lipids accumulate under the vascular endothelium, triggering a microvascular inflammatory response. This response further promotes macrophage activation, migration, and phagocytosis of lipids, thereby accelerating the formation and accumulation of foam cells and perpetuating and exacerbating the inflammatory process (Vassiliou and Farias-Pereira, 2023). The inflammatory response promotes cellular lipid uptake and accumulation and inhibits lipid metabolism, thereby exacerbating the disruption of lipid metabolism (Wang et al., 2024). In conclusion, there is a close interaction between inflammation and lipid metabolism. Abnormalities in lipid metabolism can induce inflammation, while the inflammatory response affects the homeostasis of lipid metabolism.

## 5 Conclusion

In this study, we presented a network pharmacology method that incorporated UPLC-Q-TOF-MS data from IO, target-prediction databases, and human PPI, along with animal experimental validations. We demonstrated that the crucial regulation pathways and network analysis from shared targets between IO and ORG for understanding the renal protective mechanism of IO. Our research suggested that the amelioration of IO on obesity, glycolipid metabolism, proteinuria, podocyte injury in ORG mice via its modulation on TNF signal, and triterpenoids were predicated as acting components. In summary, the network pharmacology method developed here could facilitate the mechanism discovery and active compounds prediction of IO therapy and help catalyze innovation in its clinic application.

## 6 Prospective

**FIGURE 10:**
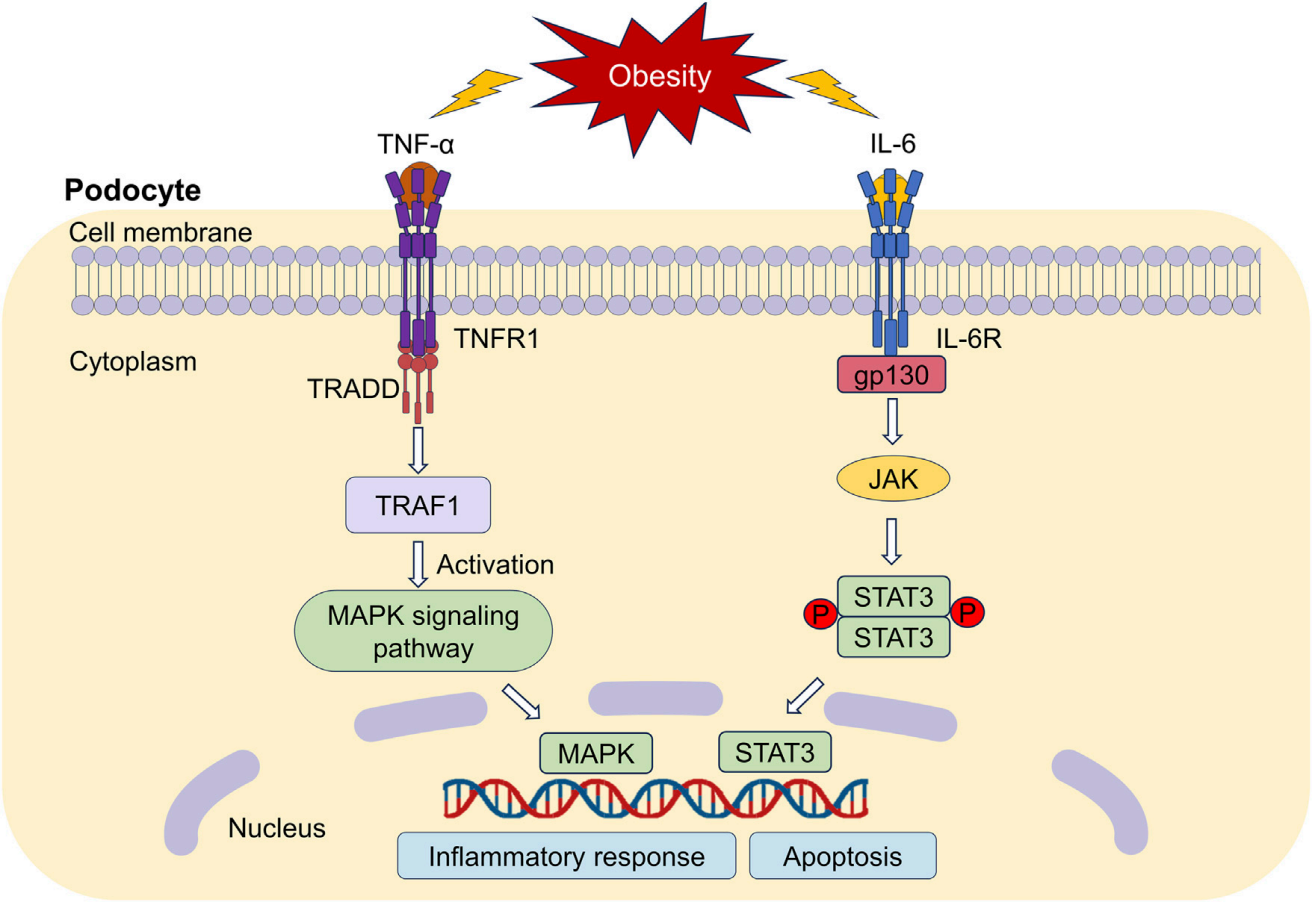
Downstream regulatory mechanisms of TNF-α and IL-6. TNFR1: TNF-α receptor 1; TRAF1: TNF receptor associated factor 1; TRADD: TNFR1-associated death domain protein; MAPK: mitogen-activated protein kinase; IL-6R: Interleukin 6 receptor; JAK: Janus-activated kinase; STAT3: signal transducer and activator of transcription 3.

Our network pharmacology studies showed that mitogen-activated protein kinase (MAPK) and STAT3 were the key targets of IO against ORG. Therefore, the perspective was centered around MAPK and STAT3 in Figure 10 based on our research on TNF-α and IL-6.

TNF-α promotes inflammation and tissue injury by binding to the TNF-α receptor 1 (TNFR1) receptor (Pesce et al., 2022). TNFR1 recognizes the signaling molecule TNF-α, which in turn activates MAPK pathway cascade and induces the activation of downstream inflammatory factors (Zhao et al., 2019), thereby damaging renal podocytes. We believed that IO might reduce inflammatory response by inhibiting MAPK signaling pathway not only in DKD (Jiang et al., 2020), but also in ORG.

Receptor proteins for IL-6 include the specific IL-6 binding receptor protein (IL-6R) and gp130, which is known as a signal transduction protein (Widjaja and Cook, 2024). IL-6R binds to gp130 could activate Janus-activated kinase and STAT3. High expression of STAT3 reduce nephrin expression, contributing to podocyte apoptosis (Lei et al., 2019). The inhibition of the IL-6/STAT3 signaling improves podocyte hypertrophy and protects podocytes (Zhou et al., 2021). We will pay our attention on the relationship between different kinds of podocyte-specific proteins and IL-6/STAT3 pathway.

At present, there are fewer reports on triterpenoid components of IO ameliorating renal inflammatory responses, but triterpenoid components, such as 3,4-dihydroxylbenzalactone, hispidin, hispolon and inotodiol from IO have been reported to inhibit TNF-induced activation of NF-κB (Zhao and Zheng, 2021).

## Data Availability

The original contributions presented in the study are included in the article/Supplementary Material, further inquiries can be directed to the corresponding authors.
