# Supplementary material for: Effects of Inonotus obliquus on ameliorating podocyte injury in ORG mice through TNF pathway and prediction of active compounds
Source: Front Pharmacol. 2024 Aug 21;15:1426917. doi: 10.3389/fphar.2024.1426917 (PMC11371614; doi:10.3389/fphar.2024.1426917)
Supplement: Supplementary file 1 [file Table1.DOCX]

Supplementary Material

# Supplementary Tables

**Supplementary Table 1. The compound identification information of IO**

| Number | Full name | m/z | Adduct | MSMS spectrum | Classification | |
| --- | --- | --- | --- | --- | --- | --- |
| 1 | Inoterpene F | 441.37 | [M+H]+ | 81.07, 215.17 | Triterpenoids | |
| 2 | Inoterpene D | 481.37 | [M+Na]+ | 463.24 | Triterpenoids | |
| 3 | Inoterpene C | 459.38 | [M+H]+ | 247.2 | Triterpenoids | |
| 4 | Inoterpene E | 497.36 | [M+H]+ | 479.25 | Triterpenoids | |
| 5 | Inonotsulide A | 471.35 | [M-H]- | 373.29 | Triterpenoids | |
| 6 | Inonotsulide B | 471.35 | [M-H]- | 128.01 | Triterpenoids | |
| 7 | Inonotsulide C | 471.35 | [M-H]- | 383.28 | Triterpenoids | |
| 8 | Inonotusol G | 457.36 | [M+H]+ | 179.14, 439.36 | Triterpenoids | |
| 9 | Inonotusol D | 487.34 | [M-H]- | 425.26, 443.28 | Triterpenoids | |
| 10 | Inonotusol E | 487.34 | [M-H]- | 443.28 | Triterpenoids | |
| 11 | Bufotalin | 467.24 | [M+Na]+ | 263.12 | Saponins | |
| 12 | Emetine | 481.31 | [M+H]+ | 283.17 | Alkaloid | |
| 13 | Uvaol | 425.37 | [M+H-H2O]+ | 201.16, 407.36 | Triterpenoids | |
| 14 | β-Ursolic acid | 455.35 | [M-H]- | 339.26, 373.27 453.90 | Triterpenoids | |
| 15 | Trametenolic acid | 457.36 | [M+H]+ | 439.36 | Triterpenoids | |
| 16 | Soyasapogenol B | 481.36 | [M+Na]+ | 463.24 | Triterpenoids | |
| 17 | α-Boswellic Acid | 479.35 | [M+Na]+ | 461.32 | / | |
| 18 | Darutigenol | 345.24 | [M+Na]+ | 327.18, | / | |
| 19 | Oleanonic Acid | 455.35 | [M-H]- | 339.26, 373.27 453.90 | Triterpenoids | |
| 20 | Inotodiol | 443.33 | [M+H]+ | 441.92 | Triterpenoids | |
| 21 | Inonotsutriol E | 481.36 | [M+Na]+ | 463.37, 479.85 | Triterpenoids | |
| 22 | Hispiduloside | 485.11 | [M+Na]+ | 467.21 | Triterpenoids | |
| 23 | Saponaceoic acid I | 495.34 | [M+Na]+ | 477.33 | Triterpenoids | |
| 24 | 23-hydroxybetulinic acid | 495.34 | [M+Na]+ | 477.33 | Triterpenoids | |
| 25 | Ursolic Acid | 455.35 | [M-H]- | 339.26, 373.27 | Triterpenoids | |
| 26 | 3β-hydroxy-lanosta-8,24-dien-21-al | 441.37 | [M+H]+ | 81.07, 423.38 | Triterpenoids | |
| 27 | Fuscoporianol C | 459.38 | [M+H]+ | 225.19 | Triterpenoids | |
| 28 | 6,8-Diprenylorobol | 423.18 | [M+H]+ | 405.35 | Triterpenoids | |
| 29 | Soyasapogenol A | 497.36 | [M+Na]+ | 243.04 | Triterpenoids | |
| 30 | Glycyrrhetic Acid | 493.33 | [M+Na]+ | 191.12, 491.7 | Triterpenoids | |
| 31 | Isokurarinone | 439.21 | [M+H]+ | 421.35 | Flavonoids | |
| 32 | **Compound 1** | 339.16 | [M+H]+ | 142.94 | Triterpenoids | |
| 33 | Vindoline | 457.23 | [M+H]+ | 120.96, 337.23 | Alkaloid | |
| 34 | Dodecylbenzenesulfonic Acid | 325.18 | [M-H]- | 183.01 | / | |
| 35 | Prunetin | 285.08 | [M+H]+ | 209.06, 257.07 267.08 | Flavonoids | |
| 36 | Pseudolaric Acid B | 455.16 | [M-H]- | 437.33 | Diterpenoids | |
| 37 | Harpagide | 365.14 | [M+H]+ | 186.06 | Terpenoids | |
| 38 | Auraptene | 299.16 | [M+H]+ | 267.02 | Carboxylic acids | |
| 39 | Ingenol | 349.20 | [M+H]+ | 287.17 | Diterpenoids | |
| 40 | Oleanolic Acid | 457.36 | [M+H]+ | 439.35 | Triterpenoids | |
| 41 | **Compound 2** | 471.35 | [M-H]- | 373.36 | / | |
| 42 | Daidzein | 255.07 | [M+H]+ | 181.07, 199.07, 227.07, 237.05 | Triterpenoids | |
| 43 | Glabridin | 347.12 | [M+Na]+ | 287.12 | Flavonoids | |
| 44 | 7-Oxodehydroabietic acid | 315.20 | [M+Na]+ | 297.26 | Diterpenoids | |
| 45 | Kahweol | 337.18 | [M+Na]+ | 309.11 | / | |
| 46 | Timosaponin A3 | 763.42 | [M+Na]+ | 227.12, 365.10 | Saponins | |
| 47 | Deoxycholic Acid | 391.28 | [M-H]- | 345.28 | / | |
| 48 | **Compound 3** | 233.15 | [M-H2O+H]+ | 159.11, 187.14 | / | |
| 49 | Corynoxine B | 407.19 | [M+Na]+ | 147.11 | Alkaloids | |
| 50 | Zinniol | 265.15 | [M-H]- | 221.15 | / | |
| 51 | **Compound 4** | 415.21 | [M+Na]+ | 369.17, 397.2 | / | |
| 52 | Harpagoside | 517.17 | [M+Na]+ | 455.24, 499.22 | Terpenoids | |
| 53 | Angeloylgomisin H | 523.23 | [M+Na]+ | 438.19, 505.28 | Coumarins | |
| 54 | Lauryl Diethanolamide | 288.25 | [M+H]+ | 270.24 | / | |
| 55 | Seconeokadsuranic Acid A | 491.31 | [M+Na]+ | 429.28, 473.31 | Terpenoids | |
| 56 | Emodin | 269.04 | [M-H]- | 225.05, 241.04 | Flavonoids | |
| 57 | **Compound 5** | 432.24 | [M+NH4]+ | 414.78 | / | |
| 58 | 5-O-Methylvisammioside | 453.17 | [M+H]+ | 319.11 | Flavonoids | |
| 59 | Quillaic Acid | 509.32 | [M+Na]+ | 491.30 | Saponins | |
| 60 | Inonotusol B | 527.33 | [M+Na]+ | 509.33 | Triterpenoids | |
| 61 | Madecassic Acid | 527.33 | [M+Na]+ | 509.33 | Triterpenoids | |
| 62 | Isoimperatorin | 269.08 | [M-H]- | 209.06 225.05 | Coumarins | |
| 63 | Piperine | 286.15 | [M+H]+ | 268.15 | Alkaloids | |
| 64 | Inonotusol C | 511.34 | [M+Na]+ | 488.17, 493.32 | Triterpenoids | |
| 65 | Alisol F | 511.34 | [M+Na]+ | 488.17, 493.32 | Triterpenoids | |
| 66 | Cocamidopropylbetaine | 343.30 | [M+H]+ | 242.04, 326.19 | Alkaloids | |
| 67 | Obacunone | 455.20 | [M+H]+ | 437.25 | Flavonoids | |
| 68 | Polyporic Acid | 291.06 | [M+H]+ | 247.07, 273.08 | Polyphenols | |
| 69 | Saikosaponin A | 825.46 | [M+FA-H]- | 779.45 | Triterpenoids | |
| 70 | Silymarin | 505.11 | [M+H]+ | 431.24, 487.24 | Flavonoids | |
| 71 | Mycophenolic Acid | 319.12 | [M-H]- | 275.12, 287.09 | Carboxylic acids | |
| 72 | Tomatidine | 438.34 | [M+Na]+ | 394.3 | Alkaloids | |
| 73 | Formononetin | 267.06 | [M-H]- | 223.05 | Flavonoids | |
| 74 | 5-Methoxypsoralen | 217.05 | [M+H]+ | 173.06 | Terpenoids | |
| 75 | Agarotetrol | 341.10 | [M+Na]+ | 295.02, 323.1 | Polyphenols |  |
| 76 | Cucurbitacin B | 559.32 | [M+H]+ | 315.16, 541.31 | Triterpenoids |  |
| 77 | Inonotusol A | 505.35 | [M+H]+ | 441.34, 461.24 487.34 | Triterpenoids |  |
| 78 | **Compound 6** | 391.21 | [M+Na]+ | 247.12, 373.21 | Alkaloids |  |
| 79 | Brucine | 395.20 | [M+H]+ | 351.2, 377.23 | Alkaloids |  |
| 80 | Sebacic Acid | 201.11 | [M-H]- | 139.12, 183.1 | Carboxylic acids |  |
| 81 | Berberine Chloride | 336.12 | [M+H]+ | 262.1, 276.11 | Alkaloids |  |
| 82 | 8-Demethyleucalyptin | 311.09 | [M-H]- | 267.06 | Polyphenols |  |
| 83 | Arnicolide C | 335.18 | [M+H]+ | 207.12, 317.06 | Terpenoids |  |
| 84 | 3β-hydroxycinnamolide | 251.16 | [M+H]+ | 187.15, 233.14 | Terpenoids |  |
| 85 | Oxyresveratrol 3'-O-Beta-D-Glucopyranoside | 407.13 | [M+H]+ | 195.06, 345.16 389.12 | Flavonoids |  |
| 86 | 6,7-Dihydrotabersonine | 339.21 | [M+H]+ | 279.16, 321.20 | Coumarins |  |
| 87 | 1,6-O,O-Diacetylbritannilactone | 351.17 | [M+H]+ | 307.19, 333.17 | Terpenoids |  |
| 88 | Cianidanol | 291.09 | [M+H]+ | 229.07, 245.11, 273.10 | Polyphenols |  |
| 89 | Eurycomanone | 431.13 | [M+Na]+ | 297.03, 413.12 | Flavonoids |  |
| 90 | Aloesin | 395.13 | [M+H]+ | 335.14, 351.14 377.14 | / |  |
| 91 | Vincamine | 355.21 | [M+H]+ | 309.16, 337.20 | Alkaloids |  |
| 92 | Sinapoyl Aldehyde | 209.08 | [M+Na-2H]- | 121.07, 165.08 | Aldehydes |  |
| 93 | Plumbagin | 187.04 | [M-H]- | 143.04, 159.04 | Flavonoids |  |
| 94 | Glycitin | 447.13 | [M+H]+ | 267.12, 429.10 | Flavonoids |  |
| 95 | Coniferyl Aldehyde | 179.07 | [M+H]+ | 119.05, 133.06 | Aldehydes |  |
| 96 | Breviscapine | 485.07 | [M+Na]+ | 467.06 | Flavonoids |  |
| 97 | Toddalolactone | 309.13 | [M+H]+ | 234.10, 265.14, 291.11 | Flavonoids |  |
| 98 | Daidzin | 417.12 | [M+H]+ | 220.04, 399.11 | Flavonoids |  |
| 99 | Leptophylloside | 447.12 | [M+Na]+ | 107.08, 429.18 | Saponins |  |
| 100 | Naringenin | 273.08 | [M+H]+ | 77.03, 213.06, 229.04 | Flavonoids |  |
| 101 | Loliolide | 197.12 | [M+H]+ | 111.09, 179.1 | Terpenoids |  |
| 102 | Ellagic Acid | 301.00 | [M-H]- | 254.99, 282.99 | Polyphenols |  |
| 103 | Vincetoxicoside B | 471.09 | [M+Na]+ | 151.03, 219.02, 453.09 | Saponins |  |
| 104 | Arctigenin 4'-O-beta-gentiobioside | 719.25 | [M+Na]+ | 493.16, 701.24 | Saponins |  |
| 105 | Syringaldehyde | 183.07 | [M+H]+ | 50.01, 139.04 | Aldehydes |  |
| 106 | (+)-Peusedanol | 265.10 | [M+H]+ | 69.03, 219.07, 247.09 | Coumarins |  |
| 107 | 4-Methyldaphnetin | 191.03 | [M-H]- | 69.03, 147.01, 173.02 | Coumarins |  |
| 108 | M-Coumaric Acid | 163.04 | [M-H]- | 119.04 | Coumarins |  |
| 109 | Mangiferin | 423.09 | [M+H]+ | 405.10 | Flavonoids |  |
| 110 | Isovitexin | 433.11 | [M+H]+ | 415.09 | Flavonoids |  |
| 111 | Vanillin | 153.06 | [M-H]- | 67.01, 109.04 | Aldehydes |  |
| 112 | 1-propan-2-yl-2,3,4,9-tetrahydro-1H-pyrido[3,4-b]indole-3-carboxylic acid | 281.14 | [M+H]+ | 217.01, 263.15 | Carboxylic acids |  |
| 113 | Norharman | 169.07 | [M-H]- | 125.09 | Alkaloids |  |
| 114 | Puerarin | 417.12 | [M+H]+ | 55.05, 399.11 | Flavonoids |  |
| 115 | 2-Phenylethyl B-D-Glucopyranoside | 307.12 | [M+Na]+ | 261.07, 289.11 | / |  |
| 116 | Bilobalide | 349.09 | [M+Na]+ | 285.04, 331.08 | Terpenoids |  |
| 117 | 3-(4-Hydroxyphenyl)Prop-2-Enoic Acid | 163.04 | [M-H]- | 107.04, 119.04, 145.04 | Carboxylic acids |  |
| 118 | Pimelic Acid | 159.07 | [M-H]- | 95.05, 97.06 | Carboxylic acids |  |
| 119 | Caffeic Acid | 179.04 | [M-H]- | 161.03, 105.04 | Carboxylic acids |  |
| 120 | Vanillic Acid | 167.03 | [M-H]- | 91.02, 123.03 | Carboxylic acids |  |
| 121 | 4-Acetoxyphenol | 151.04 | [M-H]- | 105.04 | Phenols |  |
| 122 | Esculetin | 179.03 | [M-H]- | 59.01, 93.04, 135.05 | Coumarins |  |
| 123 | Apigenin | 271.06 | [M+H]+ | 53.03, 211.09, 253.10 | Flavonoids |  |
| 124 | 2-Isopropylmalic Acid | 175.06 | [M-H]- | 115.05, 157.05 | Carboxylic acids |  |
| 125 | Mandelic Acid | 151.04 | [M-H]- | 63.02, 133.03, 136.03 | Carboxylic acids |  |
| 126 | Syringic Acid | 197.05 | [M-H]- | 95.02, 153.05 | Carboxylic acids |  |
| 127 | Protocatechuic Aldehyde | 137.02 | [M-H]- | 109.04, 119.05 | Aldehydes |  |
| 128 | Methylgallate | 183.03 | [M-H]- | 165.02 | Phenols |  |
| 129 | Maltol | 127.04 | [M+H]+ | 109.04 | / |  |
| 130 | 2,3-Dihydroxy-1-(4-Hydroxy-3-Methoxyphenyl)-Propan-1-One | 213.08 | [M+H]+ | 195.08 | / |  |
| 131 | 2,3-Dihydroxybenzoic Acid | 153.02 | [M-H]- | 91.02, 109.02 | Carboxylic acids |  |
| 132 | Catechol | 109.03 | [M-H]- | 65.00, 91.02 | Phenols |  |
| 133 | Homogentisic Acid | 167.03 | [M-H]- | 105.03, 123.03 | Carboxylic acids |  |
| 134 | 4-Methylcatechol | 123.05 | [M-H]- | 105.02 | Phenols |  |
| 135 | Phenylacetic Acid | 135.05 | [M-H]- | 79.05, 117.05 | Carboxylic acids |  |
| 136 | Matrine | 249.20 | [M+H]+ | 175.03, 231.02 | Alkaloids |  |
| 137 | Itaconate | 129.02 | [M-H]- | 85.02 | Carboxylic acids |  |
| 138 | Gallic Acid | 169.01 | [M-H]- | 107.02, 125.02, 151.00 | Carboxylic acids |  |
| 139 | 4-Quinolinecarboxylic Acid | 174.05 | [M-H]- | 112.04, 130.06 | Carboxylic acids |  |
| 140 | Succinic Acid | 117.02 | [M-H]- | 73.04, 99.02 | Carboxylic acids |  |
| 141 | L-Pyroglutamic Acid | 130.05 | [M+H]+ | 56.05, 84.05 | Carboxylic acids |  |
| 142 | Citric Acid | 191.02 | [M-H]- | 129.02 | Carboxylic acids |  |
| 143 | Nicotinic Acid | 124.04 | [M+H]+ | 50.01, 80.04, 106.02 | Vitamins |  |
| 144 | Nicotinamide | 123.05 | [M-H]- | 93.03, 121.02 | Vitamins |  |
| 145 | (S)-Malate | 133.01 | [M-H]- | 59.01, 71.00, 114.99 | Carboxylic acids |  |
| 146 | Betaine | 118.09 | [M+H]+ | 59.09 | Alkaloids |  |

**Note:** Compound 1: 9-[(3,7-Dimethyl-2,6-octadien-1-yl)oxy]-7H-furo[3,2-g][1]benzopyran-7-one. Compound 2: (E,6S)-7-Hydroxy-2-Methyl-6-[(10S,13S,14S,17S)-4,4,10,13,14-Pentamethyl-3-Oxo-1,2,5,6,7,11,12,15,16,17-Decahydrocyclopenta[A]Phenanthren-17-Yl]Hept-2-Enoic Acid. Compound 3: (4aR,5S,8aS,9aR)-9a-hydroxy-3,4a,5-trimethyl-5,6,7,8,8a,9-hexahydro-4H-benzo[f][1]benzofuran-2-one. Compound 4: (1S,2R,4Ar,8Ar)-1-Acetoxy-7-Isopropylidene-1,4A-Dimethyl-6-Oxodecahydro-2-Naphthalenyl 2,3-Dimethyl-2-Oxiranecarboxylate. Compound 5: 7B,9-Dihydroxy-3-(Hydroxymethyl)-1,1,6,8-Tetramethyl-5-Oxo-1,1A,1B,4,4A,5,7A,7B,8,9-Decahydro-9Ah-Cyclopropa[3,4]Benzo[1,2-E]Azulen-9A-Yl Acetate. Compound 6: 5-(3-methoxyphenyl)-N-(octahydro-2H-quinolizin-1-ylmethyl)-1H-pyrazole-3-carboxamide.
