# Supplementary material for: Effects of Inonotus obliquus on ameliorating podocyte injury in ORG mice through TNF pathway and prediction of active compounds
Source: Front Pharmacol. 2024 Aug 21;15:1426917. doi: 10.3389/fphar.2024.1426917 (PMC11371614; doi:10.3389/fphar.2024.1426917)
Supplement: Supplementary file 2 [file Table2.DOCX]

Supplementary Material

# Supplementary Tables

**Supplementary Table 2. The core targets of IO against ORG.**

| No. | Gene name | Degree | No. | Gene name | Degree |
| --- | --- | --- | --- | --- | --- |
|  | IL6 | 108 | 17 | REN | 50 |
|  | TNF | 106 | 18 | RELA | 48 |
|  | AKT1 | 100 | 19 | ACE2 | 48 |
|  | STAT3 | 90 | 20 | GCG | 48 |
|  | PPARG | 84 | 21 | NR3C1 | 44 |
|  | CASP3 | 82 | 22 | PIK3R1 | 42 |
|  | TLR4 | 74 | 23 | CASP8 | 42 |
|  | ICAM1 | 70 | 24 | AR | 42 |
|  | SIRT1 | 68 | 25 | DPP4 | 42 |
|  | SERPINE1 | 64 | 26 | HMOX1 | 42 |
|  | PTGS2 | 62 | 27 | FLT1 | 40 |
|  | VCAM1 | 58 | 28 | NLRP3 | 40 |
|  | MAPK1 | 56 | 29 | JAK1 | 36 |
|  | ACE | 56 | 30 | CCR5 | 36 |
|  | CCND1 | 52 | 31 | SELP | 36 |
|  | NOS3 | 52 |  |  |  |
