# Supplementary material for: Effects of Inonotus obliquus on ameliorating podocyte injury in ORG mice through TNF pathway and prediction of active compounds
Source: Front Pharmacol. 2024 Aug 21;15:1426917. doi: 10.3389/fphar.2024.1426917 (PMC11371614; doi:10.3389/fphar.2024.1426917)
Supplement: Supplementary file 3 [file Table3.DOCX]

Supplementary Material

# Supplementary Tables

**Supplementary Table 3. Components of IO absorbed into blood**

| No. | Full name | m/z | Classification | Source | No. | Full name | m/z | Classification | Source |
| --- | --- | --- | --- | --- | --- | --- | --- | --- | --- |
| 1 | Harpagoside | 517.17 | Triterpenoids | prototype | 28 | 3β-hydroxycinnamolide | 441.37 | / | prototype |
| 2 | Deoxycholic Acid | 471.35 | / | prototype | 29 | Matrine | 432.24 | Alkaloid | prototype |
| 3 | Inonotsulide B | 471.35 | Triterpenoids | prototype | 30 | 5-Methoxypsoralen | 415.21 | Terpenoids | prototype |
| 4 | Inonotsulide A | 471.35 | Triterpenoids | prototype | 31 | Sebacic Acid | 395.20 | Carboxylic acids | prototype |
| 5 | Inonotsulide C | 467.24 | Triterpenoids | prototype | 32 | Citric acid | 351.18 | Carboxylic acids | prototype |
| 6 | Bufotalin | 457.36 | Saponins | prototype | 33 | Plumbagin | 349.20 | Flavonoids | prototype |
| 7 | Trametenolic acid | 457.36 | Triterpenoids | prototype | 34 | Syringaldehyde | 345.24 | Aldehydes | prototype |
| 8 | Inonotusol G | 457.36 | Triterpenoids | prototype | 35 | Homogentisic Acid | 343.30 | Carboxylic acids | prototype |
| 9 | Oleanolic Acid | 441.37 | Triterpenoids | prototype | 36 | Vanillic Acid | 339.16 | Carboxylic acids | prototype |
| 10 | Inotodiol | 443.33 | Triterpenoids | prototype | 37 | M-Coumaric Acid | 319.12 | Coumarins | prototype |
| 11 | Inoterpene F | 441.37 | Triterpenoids | prototype | 38 | 3-(4-Hydroxyphenyl)Prop-2-Enoic Acid | 309.13 | Carboxylic acids | prototype |
| 12 | 3β-hydroxy-lanosta-8,24-dien-21-al | 432.24 | Triterpenoids | prototype | 39 | (S)-Malate | 269.08 | Carboxylic acids | prototype |
| 13 | **Compound 5** | 415.21 | / | prototype | 40 | L-Pyroglutamic Acid | 269.05 | Carboxylic acids | prototype |
| 14 | **Compound 4** | 395.20 | / | prototype | 41 | Betaine | 265.15 | Alkaloid | prototype |
| 15 | Brucine | 391.28 | Alkaloid | prototype | 42 | Succinic Acid | 265.11 | Carboxylic acids | prototype |
| 16 | 1,6-O,O-Diacetylbritannilactone | 351.17 | Terpenoids | prototype | 43 | Catechol | 255.07 | Phenols | prototype |
| 17 | Ingenol | 349.20 | Diterpenoids | prototype | 44 | Taurodeoxycholic acid | 500.30 |  | prototype |
| 18 | Darutigenol | 517.17 | / | prototype | 45 | Taurocholic acid | 498.29 |  | metabolites |
| 19 | Cocamidopropylbetaine | 500.30 | Alkaloid | prototype | 46 | Isosakuranin | 471.13 |  | metabolites |
| 20 | **Compound 1** | 471.35 | Triterpenoids | prototype | 47 | N-Acetylphenylalanine | 208.10 |  | metabolites |
| 21 | Mycophenolic Acid | 471.35 | Carboxylic acids | prototype | 48 | Theophylline | 203.05 |  | metabolites |
| 22 | Toddalolactone | 471.35 | Flavonoids | prototype | 49 | 3-Hydroxycinnamic acid | 193.05 |  | metabolites |
| 23 | Isoimperatorin | 467.24 | Coumarins | prototype | 50 | 4-Methoxycinnamic acid | 177.05 |  | metabolites |
| 24 | Emodin | 457.37 | Flavonoids | prototype | 51 | 4-aminovaleric acid betaine | 160.13 |  | metabolites |
| 25 | Zinniol | 457.36 | / | prototype | 52 | 4-Hydroxyquinoline | 144.04 |  | metabolites |
| 26 | (+)-Peusedanol | 457.36 | Coumarins | prototype | 53 | D-(+)-Malic acid | 133.01 |  | metabolites |
| 27 | Daidzein | 441.37 | Triterpenoids | prototype |  |  |  |  |  |

**Note:** Compound 1: 9-[(3,7-Dimethyl-2,6-octadien-1-yl)oxy]-7H-furo[3,2-g][1]benzopyran-7-one. Compound 4: (1S,2R,4Ar,8Ar)-1-Acetoxy-7-Isopropylidene-1,4A-Dimethyl-6-Oxodecahydro-2-Naphthalenyl 2,3-Dimethyl-2-Oxiranecarboxylate. Compound 5: 7B,9-Dihydroxy-3-(Hydroxymethyl)-1,1,6,8-Tetramethyl-5-Oxo-1,1A,1B,4,4A,5,7A,7B,8,9-Decahydro-9Ah-Cyclopropa[3,4]Benzo[1,2-E]Azulen-9A-Yl Acetate.
