# Supplementary material for: Effects of Inonotus obliquus on ameliorating podocyte injury in ORG mice through TNF pathway and prediction of active compounds
Source: Front Pharmacol. 2024 Aug 21;15:1426917. doi: 10.3389/fphar.2024.1426917 (PMC11371614; doi:10.3389/fphar.2024.1426917)
Supplement: Supplementary file 4 [file DataSheet1.DOCX]

Supplementary Material

# Supplementary Method

## Measurement of intraperitoneal glucose tolerance test (IPGTT) and insulin tolerance test (IPITT)

IPGTT and IPITT experiments were performed after 10 weeks of Inonotus obliquus intervention in the obesity model mice. In the IPGTT experiment, the mice were fasted for 12 h at night and then injected intraperitoneally with 10% glucose solution (2 g/kg). In the IPITT experiment, the mice were fasted for 6 h and then injected intraperitoneally with insulin solution (0.75 U/kg). Blood was collected from the tail vein of the mice at 0, 30, 60, 90, and 120 min after intraperitoneal injection. Blood glucose values were measured and plotted on a line graph, and the area under the curve was then calculated.

# Supplementary Figures and Tables

##
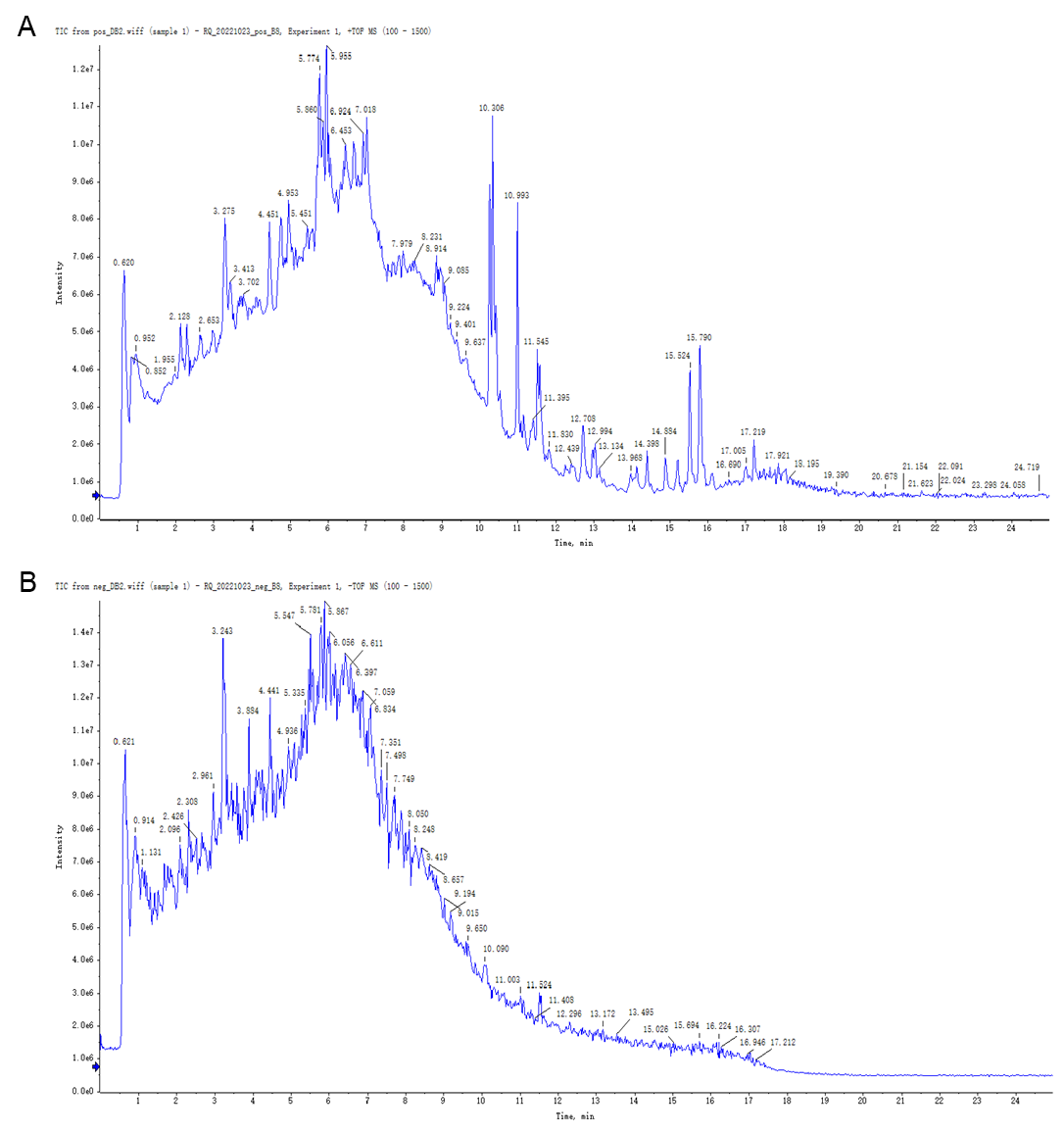
Supplementary Figures

**Supplementary Figure 1.** Analysis of *Inonotus obliquus* extracts by UPLC-MS/MS profiling. (A) Total ion chromatogram in positive mode. (B) Total ion chromatogram in negative mode.


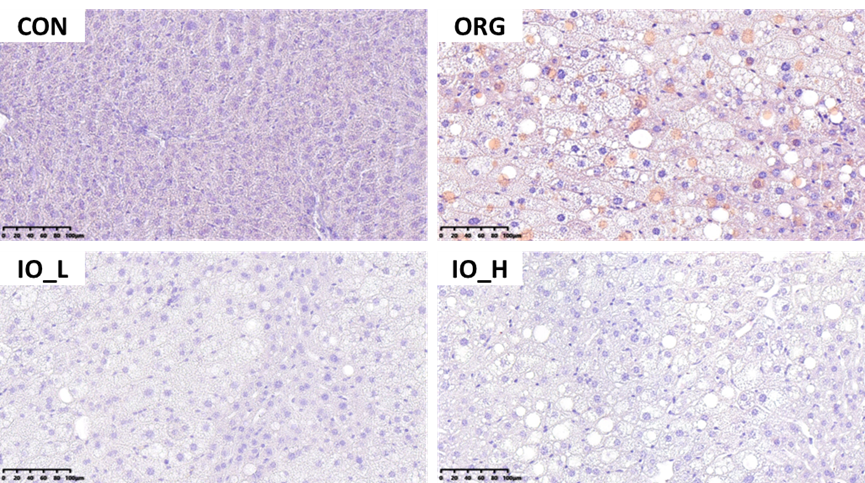
**Supplementary Figure 2.** Effects of IO on liver lipid deposition in ORG mice (Oil red O staining × 200). CON: control; ORG: obesity-related glomerulopathy; IO_L: the model group treated with *Inonotus obliquus* (75 mg/kg/day); IO_H: the model group treated with *I. obliquus* (150 mg/kg/day).


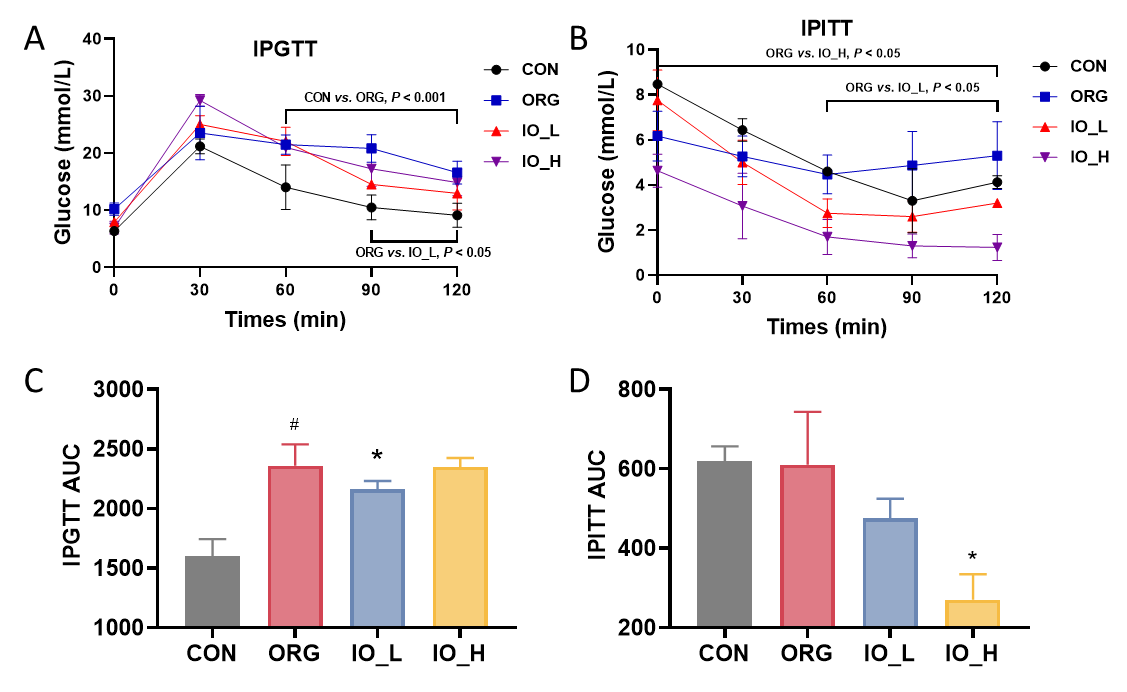
**Supplementary Figure 3.** Effects of IO on IPGTT and IPITT in ORG mice. (A) Levels of IPGTT. (B) Levels of IPITT. (C) AUC of IPGTT. (D) AUC of IPITT. IPGTT: intraperitoneal glucose tolerance test; IPITT: intraperitoneal insulin tolerance test; AUC: area under curve; CON: control; ORG: obesity-related glomerulopathy; IO_L: the model group treated with *Inonotus obliquus* (75 mg/kg/day); IO_H: the model group treated with *I. obliquus* (150 mg/kg/day). Data are presented as mean ± SD. ^#^*P* < 0.05 vs. CON; ^*^*P* < 0.05 vs. ORG.


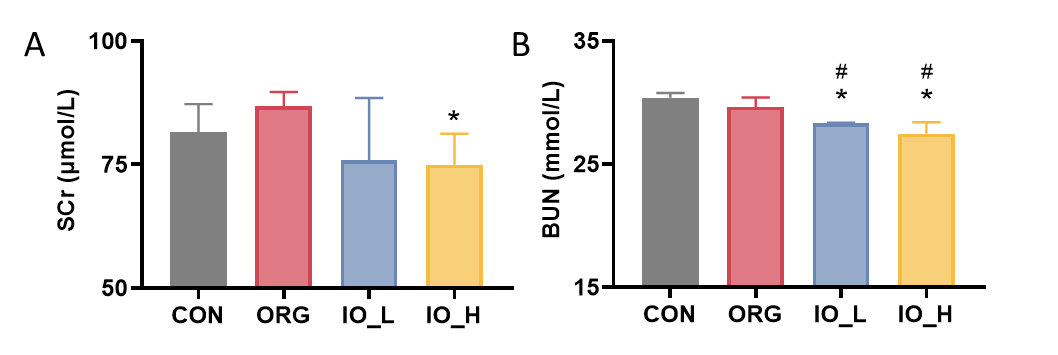
**Supplementary Figure 4.** Effects of IO on renal function in ORG mice. (A) Levels of SCr. (B) Levels of BUN. SCr: serum creatinine; BUN: blood urea nitrogen; CON: control group; ORG: obesity-related glomerulopathy group; IO_L: the model group treated with *Inonotus obliquus* (75 mg/kg/day); IO_H: the model group treated with *I. obliquus* (150 mg/kg/day). Data are presented as mean ± SD. ^#^*P* < 0.05 vs. CON; ^*^*P* < 0.05 vs. ORG.

**
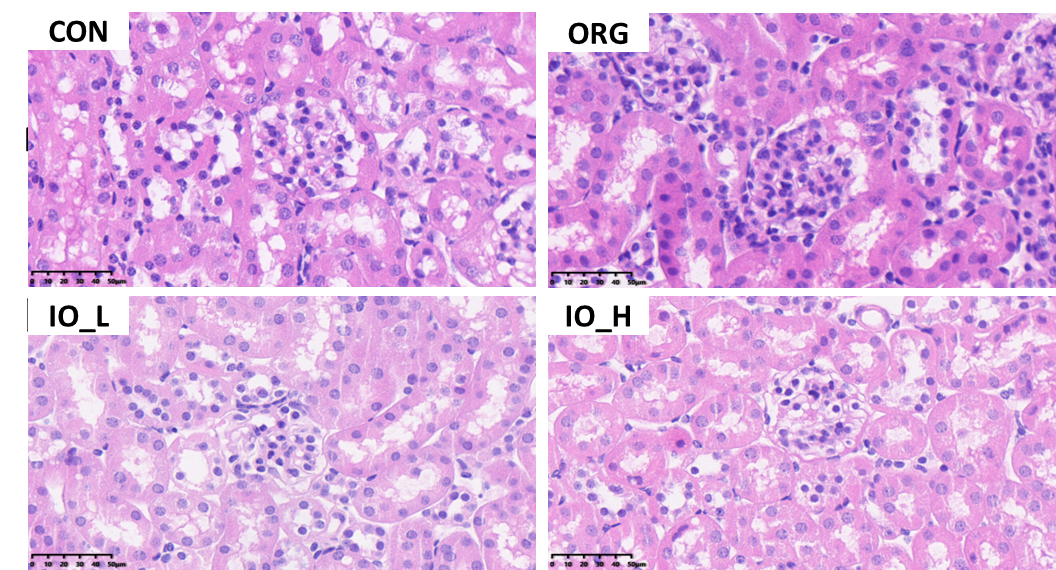
Supplementary Figure 5.** Effects of IO on glomerulus morphology in ORG mice (HE staining × 200). CON: control group; ORG: obesity-related glomerulopathy group; IO_L: the model group treated with *Inonotus obliquus* (75 mg/kg/day); IO_H: the model group treated with *I. obliquus* (150 mg/kg/day). Data are presented as mean ± SD. ^#^*P* < 0.05 vs. CON; ^*^*P* < 0.05 vs. ORG.


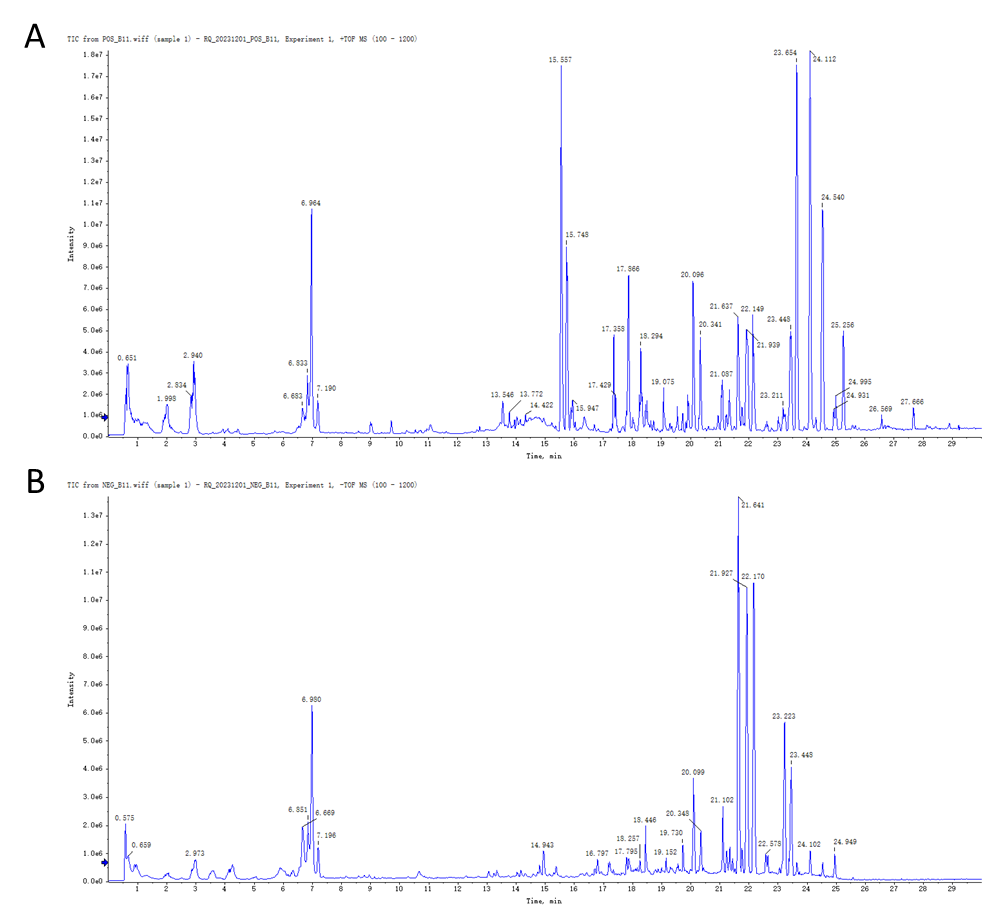
**Supplementary Figure 6.** Analysis of animal serum by UPLC-MS/MS profiling. (A) Total ion chromatogram in positive mode. (B) Total ion chromatogram in negative mode.
